# Lipid-Centric Approaches in Combating Infectious Diseases: Antibacterials, Antifungals and Antivirals with Lipid-Associated Mechanisms of Action

**DOI:** 10.3390/antibiotics12121716

**Published:** 2023-12-11

**Authors:** Olga S. Ostroumova, Svetlana S. Efimova

**Affiliations:** Laboratory of Membrane and Ion Channel Modeling, Institute of Cytology, Russian Academy of Sciences, Tikhoretsky Ave. 4, St. Petersburg 194064, Russia; efimova@incras.ru

**Keywords:** membrane, lipid biosynthesis, antimicrobials, antibiotics, drug resistance, pore formation, ion channels, membrane curvature stress

## Abstract

One of the global challenges of the 21st century is the increase in mortality from infectious diseases against the backdrop of the spread of antibiotic-resistant pathogenic microorganisms. In this regard, it is worth targeting antibacterials towards the membranes of pathogens that are quite conservative and not amenable to elimination. This review is an attempt to critically analyze the possibilities of targeting antimicrobial agents towards enzymes involved in pathogen lipid biosynthesis or towards bacterial, fungal, and viral lipid membranes, to increase the permeability via pore formation and to modulate the membranes’ properties in a manner that makes them incompatible with the pathogen’s life cycle. This review discusses the advantages and disadvantages of each approach in the search for highly effective but nontoxic antimicrobial agents. Examples of compounds with a proven molecular mechanism of action are presented, and the types of the most promising pharmacophores for further research and the improvement of the characteristics of antibiotics are discussed. The strategies that pathogens use for survival in terms of modulating the lipid composition and physical properties of the membrane, achieving a balance between resistance to antibiotics and the ability to facilitate all necessary transport and signaling processes, are also considered.

## 1. Introduction

Here, we provide an overview of antibacterial, antifungal, and antiviral agents that target lipid biosynthesis and modulate the properties of pathogen membranes, including pore formation, the induction of curvature stress, and full disruption. Taking into account the emphasis on the lipid-associated mechanisms of action of potent antimicrobial drugs, to compare various agents that inhibit lipid biosynthesis, the concentrations causing a twofold decrease in the activity of appropriate enzymes are presented. The threshold concentration in the membrane bathing solution is chosen to quantitatively characterize the effectiveness of various pore-forming antibiotics. The modulation of the properties of the host cell plasma membranes, targeting enzymes engaged in the regulation of lipid metabolism and the biosynthesis of pathogens’ cell wall components, are not reviewed.

## 2. Antibacterials with Lipid-Associated Mechanisms of Action

We focus on two modes of targeting antibacterial agents towards pathogen membranes: (i) indirect action via inhibition of the biosynthesis of membrane lipids; (ii) primary interaction with lipids, which results in the disruption of the functioning of bacterial membranes. These fundamentally different possibilities are considered below.

### 2.1. Inhibitors of Membrane Lipid Biosynthesis in Bacteria

Despite the fact that bacterial cell wall biosynthesis inhibitors, especially β-lactams and glycopeptide antibiotics, inhibiting the synthesis of the peptidoglycan layer, are the most effective and extensively used classes of antibiotics [1,2], they are not covered in this work, which focuses on targeting the lipid membrane. This part of the review concerns the key enzymes in the biosynthesis of membrane lipids in bacteria and their inhibitors. Possible ways to regulate the biosynthesis, transport, and degradation of lipids are not considered.

#### 2.1.1. Biosynthesis of Fatty Acids of Bacterial Membrane Lipids

**Figure 1:**
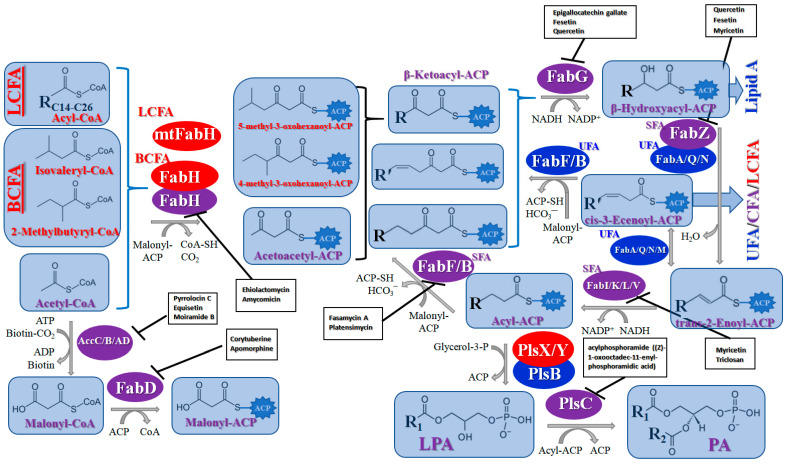
Schematic representation of the synthesis of fatty acids of bacterial membrane lipids. The red, blue, and purple ellipses indicate that the enzyme is produced by Gram-positive, Gram-negative, or both Gram-positive and Gram-negative bacteria, respectively. Some examples of enzyme inhibitors are shown in the black box. Abbreviations: **AccC**—biotin carboxylase; **AccB**—biotin carboxyl carrier protein; **AccAD**—biotin carboxyl transferase; **FabD**—malonyl-CoA:acyl carrier protein (ACP) transacylase; **FabH**, **FabF**, and **FabB**—β-ketoacyl-ACP synthase III (KAS III), II (KAS II), and I (KAS I), respectively; **mtFabH**—FabH homolog of *Mycobacterium tuberculosis*; **FabG**—NADPH-dependent β-ketoacyl-ACP reductase; **FabZ**—β-hydroxyacyl-ACP dehydratase; **FabA**/**FabQ**/**FabN**—bifunctional β-hydroxyacyl-ACP dehydratase/*trans*-2-,*cis*-3-decenoyl-ACP isomerase; **FabM**—*trans*-2-,*cis*-3-decenoyl-ACP isomerase; **FabI**/**FabK**/**FabL**/**FabV**—*trans*-2-enoyl-ACP reductase; **PlsX**—phosphate acyltransferase; **PlsY**—acyl-phosphate:glycerol-3-phosphate acyltransferase; **PlsB**—glycerol-3-phosphate acyltransferase; **PlsC**—1-acyl-sn-glycerol-3-phosphate acyltransferase; BCFA—branched-chain fatty acids; LCFA—long-chain fatty acids; LPA—lysophosphatidic acid; PA—phosphatidic acid; UFA—unsaturated fatty acids; SFA—saturated fatty acids; CFA—cyclopropane fatty acids; R, R′, R_1_, and R_2_—fatty acid hydrocarbon radicals.

The search for selective inhibitors of enzymes participating in bacterial pathways for lipid biosynthesis is a good strategy to find novel antibiotics due to the fact that a certain lipid composition of the bacterial membrane is necessary for its proper functioning, and there is a difference in the principal organization of lipid biosynthesis in bacteria and mammals. In particular, the membrane’s fatty acid composition is very important for metabolic plasticity and the growth rate of bacteria. Bacterial and mammalian fatty acid synthases (FAS) have various types. Bacterial and plant fatty acid synthases belong to type II (FASII), and each reaction is catalyzed by distinct single-functional small proteins. Type I fatty acid synthases (FASI), present in mammals and yeast, are composed of one polypeptide chain, and each stage of FAS is accomplished by a various functional domain of this multidomain protein. Figure 1 summarizes the information about the key enzymes of FASII in different bacteria. Due to another principal organization of mammalian FASI, the specific inhibitors of key enzymes of bacterial FASII are expected to be good candidates for the development of low-toxicity antibacterials. Molecules that effectively inhibit fatty acid synthesis in bacteria are discussed extensively in the text below. Table 1 provides information on some specific inhibitors of each known enzyme, as well as their inhibitory concentrations. A demonstration of the ability of the compound to inhibit the activity of the appropriate enzyme in in vitro tests can be considered as direct evidence in favor of a lipid synthesis-related mechanism of action and a specific molecular target. For this reason, the table presents only those inhibitors for which such information can be found in the available literature. The minimum inhibitory concentrations against various bacteria are not presented in Table 1 due to the high variability depending on the bacterial strain. Examples of the most common/known inhibitors are also shown in Figure 1 and marked with a black box. Next, we analyze the possibility of pharmacologically influencing this bacterial pathway and discuss more promising chemical scaffolds for further optimization. It should be taken into account that the therapeutic strategy, in addition to analyzing the inhibitory concentrations, must take into account the risks of developing resistance to the antibiotic and the side effects of its application.

The acetyl-CoA carboxylase is represented by a multiprotein complex, containing biotin carboxylase (**AccC**), the biotin carboxyl carrier protein (**AccB**), and biotin carboxyl transferase (**AccAD**), and performing the carboxylation of acetyl-CoA to generate malonyl-CoA (Figure 1) [36]. Via virtual screening and optimization of the small molecule library, antibacterials *aminooxazoles* and *benzimidazoles* were identified as potential **AccC** inhibitors (Table 1) [3,4]. Comparing the IC_50_ values, the optimization of the *benzimidazole* scaffold seems to be a more promising way to find more potent **AccC** inhibitors, but the appropriate toxicity tests should be carried out to estimate the safe therapeutic window. The biotin carboxylation step is also known to be inhibited by *pyrrolocin C* and *equisetin* [37]. The selectivity of *pyrrolocin C* and *equisetin* between bacterial and human cells does not exceed 10 [37], and the mechanisms of their toxic action should be elucidated to increase the therapeutic window for more potent **AccC** inhibitors.

The broad-spectrum antibacterial activity of *pyrrolidinedione* derivatives, particularly *moiramide B* and *andrimid*, is referred to as targeting **AccAD** (Table 1) [5,38,39,40]. An in silico evaluation of *andrimid* showed no systemic toxicity [41]. However, bacterial resistance to *andrimid* arising from a single amino acid mutation in **AccAD** was found [42]. A promising approach, including the development of dual-use conjugated inhibitors of acetyl-CoA carboxylase composed of covalently linked motifs of *aminooxazole* (interacting with **AccC**) and *moiramide B* (targeting **AccAD**), was proposed to lower the frequency of strain resistance [43]. Preliminary studies on the antibacterial mechanism of *yanglingmycin* exhibited the potent inhibition of **AccAD**, which led to fatty acid and lipid biosynthesis being blocked, and, as a result, cell membrane destruction [44]. Among herbicides, several *haloxyfop* derivatives were found to demonstrate antimycobacterial activity via the inhibition of **AccAD** [45]. 

A malonyl-CoA:acyl carrier protein (ACP) transacylase (**FabD**) transfers the malonate group from malonyl-CoA to ACP (Figure 1). It is known that **FabD** is the target for the antibacterial action of *aporphine* alkaloids [46].

The malonyl-ACP, produced by **FabD,** is used by several β-ketoacyl-ACP synthases (KASs) of FASII: KAS I (**FabB**), KAS II (**FabF**), and KAS III (**FabH**). **FabH** initiates the cycle of elongation by condensing malonyl-ACP and acyl-CoA (Figure 1). The origin of the latter strongly depends on the bacteria. The huge variety in the fatty acid profile produced by different bacteria is defined by the substrate specificity of **FabH**. **FabH** of *Escherichia coli* is most specific for acetyl-CoA and propionyl-CoA and incapable of using branched-chain substrates and longer straight-chain acyl-CoA [47,48]. The specificity of *Streptococcus pneumoniae* **FabH** is significantly higher towards short (C_2_–C_4_) straight-chain than for branched-chain acyl-CoAs [6]. Rather than synthesizing unsaturated fatty acids (UFA) to fluidify the membranes (as Gram-negative bacteria and streptococci do), a number of Gram-positive bacteria (particularly *Bacillus subtilis* and *Staphylococcus aureus*) synthesize branched-chain fatty acids (BCFA) [2]. *B. subtilis* **FabH** displays higher efficiency with straight-chain and branched-chain acyl-CoA composed of C_4_–C_8_ compared to acetyl-CoA [48]. The substrate binding pocket of *S. aureus* **FabH** is substantially larger than that of *E. coli* FabH, and the activity of *S. aureus* **FabH** to elongate different acyl-CoAs decreases in the following order: isobutyryl → hexanoyl → butyryl → isovaleryl → acetyl-CoA [49]. The **FabH** homolog of *M. tuberculosis*, **mtFabH**, to synthesize long-chain fatty acids (LCFA) prefers long-chain acyl-CoA substrates composed of C_10_–C_16_ rather than acetyl-CoA, short-chain, or branched-chain primers, due to the long internal acyl-binding channel [7,50]. Many **FabH** inhibitors have been discovered [51], and the dramatic variance in their activity against **FabH** from various stains clearly indicates the structural differences in the protein active sites. *Thiolactomycin* is known to inhibit BCFA and straight-chain fatty acid biosynthesis by targeting the **FabH** of *Streptomyces collinus* and *Streptomyces glaucescens* [52,53]. *E. coli* and *S. pneumoniae* FabH are weakly inhibited by *thiolactomycin*, while the indole compound *SB418011* significantly inhibits *E. coli*, *S. pneumoniae*, and *Haemophilus influenza* FabH (Table 1) [6]. A number of promising inhibitors of *E.coli* **FabH** have been found, including *thiazolidine*, *chrysin*, *thiazole*, *deoxybenzoin*, *salicylaldehyde*, *pyrazole*, *cinnamate*, *carbamate*, *benzaldehyde*, *o-benzylhydroxylamine*, *vanillic acylhydrazone*, *nitroimidazole*, *pyrazoline*, and *piperidine* derivatives, *furoxan/sulfonylhydrazone* hybrids, and others [54,55,56,57,58,59,60,61,62,63,64,65,66,67,68,69,70,71,72,73,74,75]. *S. aureus* **FabH** is weakly suppressed by *thiolactomycin* and is efficiently inhibited by different *1,2-dithiole-3-ones*, *1,3,5-oxadiazin-2-ones*, and *amycomicin* [76,77,78]. Selected *benzoylaminobenzoic acid* derivatives are also potent against **FabH** of *Enterococcus faecalis* and *Streptococcus pyogenes*, demonstrate only moderate activity against *S. aureus* **FabH**, and are ineffective against *H. influenzae* **FabH** [79,80,81]. *Alkylsulfonyl* compounds and *pyrrole-2-carboxylic acid* derivatives are specific inhibitors of **mtFabH** [82,83,84]. Analyzing Table 1, one can conclude that *SB418011* has the lowest IC_50_ against **FabH** among the presented chemicals. It should be also noted that it did not demonstrate inhibitory activity against human FAS at 200-times higher concentrations [6]. Despite promising differences in selectivity, the in vivo efficacy and toxicity of *SB418011* should be evaluated in further experiments.

Two other elongating KASs, **FabB** and **FabF**, operating later in the cycle, use acyl-ACP as the substrate for subsequent condensations, instead of the acetyl-CoA used by **FabH** [85] (Figure 1). The structural similarities between the active sites of **FabB**, **FabF**, and **FabH** [86,87,88,89] presume the development of antimicrobials that are able to hit several KASs at the same time, preventing the de novo synthesis of the fatty acids required for bacterial growth and survival. Two fungal products, *thiolactomycin* and *cerulenin*, are non-selective inhibitors of KASs, blocking **FabB**, **FabF**, and **FabH** with varying degrees of success [8,9,90,91]. It is found that *cerulenin* is characterized by its preferential selectivity towards **FabB** and **FabF** and is a very poor inhibitor of **FabH** (Table 1) [6,7,8,76]. The alteration in the inhibitory activity of *cerulenin* against various KASs is suggested to be a result of differences in the catalytic triads of the β-ketoacyl-ACP synthases and the size of the acyl-chain binding pockets [90]. Natural bacterial diterpenoid products *platensimycin* and *platencin* also target the KASs of FASII [92]. *Platensimycin* preferentially inhibits the chain elongation enzyme **FabF**, whereas *platencin* inhibits both chain initiation and elongation-condensing KASs, **FabH** and **FabF** (Table 1) [10,11,12,93,94,95,96]. According to the IC_50_ information presented in Table 1, *platensimycin* is the most potent inhibitor of **FabF**. Moreover, it has great potential to inhibit methicillin-resistant *Staphylococcus aureus* and vancomycin-resistant *Enterococci* [97]. The low mammalian cell toxicity and the lack of antifungal activity indicate that *platensimycin* acts selectively [12,98]. This makes it extremely promising to search for new *platensimycin*-based antimicrobials in order to improve its pure pharmacokinetic properties. Thus, several semisynthetic analogs of *platensimycin* with enhanced in vivo efficacy towards MRSA infection in a mouse peritonitis model and improved pharmacokinetic properties have been developed [99,100]. In silico docking studies clearly demonstrated that the specific structural motif presented in *fasamycins* is predicted to be one more naturally occurring pharmacophore for the specific inhibition of **FabF** [101]. A series of *N-substituted benzoxazolinones* were also shown to be active towards elongating KASs, **FabB** and **FabF**, by docking into the *thiolactomycin* binding site [102]. 

An NADPH-dependent β-ketoacyl-ACP reductase (**FabG**) produces the reduction of β-ketoacyl-ACP to β-hydroxyacyl-ACP. β-hydroxyacyl-ACP dehydrases (**FabZ** or **FabA**) and dehydrates β-hydroxyacyl-ACP to yield *trans*-2-enoyl-ACP. Another NADPH-dependent reductase of the FASII, *trans*-2-enoyl-ACP reductase (**FabI**), reduces *trans*-2-enoyl-ACP to form acyl-ACP, which can reenter the elongation cycle as a substrate for elongating KASs, **FabB** or **FabF**, or can be used for lipid production via phospholipid acyltransferases (**PlsB**/**PlsC** or **PlsX**/**PlsY**/**PlsC** system) [36] (Figure 1). 

A broad range of plant polyphenols, including epigallocatechin gallate, gallocatechin gallate, epicatechin gallate, catechin gallate, luteolin-7-O-glucoside, luteolin, quercetin, fisetin, morin, and myricetin, are potent inhibitors of all three crucial FASII enzymes, **FabG**, **FabZ**, and **FabI** (Table 1) [13,14,16]. Hexachlorophene and its anthelmintic bis-(2-hydroxyphenyl)methane/sulfide analogs are believed to exhibit antimalarial activity by inhibiting *Plasmodium falciparum* **FabG** [103]. Trans-cinnamic acid derivatives and macrolactins showed the inhibition of *E. coli* and *S. aureus* **FabG** (Table 1) [17,104]. Ethyl-6-bromo-2-((dimethylamino)methyl)-5-hydroxy-1-phenyl-1H-indole-3-carboxylate was demonstrated to be potent against Acinetobacter *baumannii* **FabG** [105]. A series of small-molecule *Pseudomonas aeruginosa* **FabG** inhibitors were identified [106]. According to Table 1, various catechin gallates demonstrate the most impressive activity (expressed as the lowest levels of IC_50_) against three key FASII enzymes at once, compared to other inhibitors presented. The triple inhibiting action of these polyphenols, as well as their relatively low toxicity, has kept these naturally occurring compounds as the focus of researchers’ attention in terms of finding more safe antibiotics. For example, a safe intake level of green tea polyphenol epigallocatechin gallate, derived from toxicological and human safety data, is about 300 mg per day for adults [107]. We find another approach that involves combining natural polyphenols with other antibiotics that have alternative mechanisms of action on pathogen metabolism to be the most relevant [108,109,110].

**FabZ** is a crucial enzyme to elongate both saturated fatty acids (SFA) and UFA, and this is why it is an attractive target for the discovery of new antibacterials. The inhibitors of *P. falciparum* **FabZ**, *NAS-21* (4,4,4-trifluoro-1-(4-nitrophenyl)-butane-1,3-dione), *NAS-91* (4-chloro-2-[(5-chloroquinolin-8-5 yl)oxyl]phenol), and their variants, were identified [111]. *NAS-21* and *NAS-91* analogs also demonstrated activity against mycobacterial **FabZ** (Table 1) [18]. The natural anthraquinone *emodin* (3-methyl-1,6,8-trihydroxyanthraquinone) and several synthetic inhibitors of *Helicobacter pylori* **FabZ**, based on two promising chemical scaffolds, namely (*3,5-dibromo-2,4-dihydroxy-benzylidene)-hydrazide* and *2-chloro-5-{5-[3-(2-methoxy-ethyl)-4-oxo-2-phenylimino-thiazolidin-5-ylidenemethyl]-furan-2-yl}-benzoic acid*, were discovered (Table 1) [20,112]. Two novel inhibitors of *Francisella tularensis* and *Yersinia pestis* **FabZ**, *mangostin* and *stictic acid*, have been found (Table 1) [19]. Moreover, *1,4-naphthoquinone* and *juglone* (5-hydroxyl-1,4-naphthoquinone) is a dual inhibitor of **FabZ** and **FabD** that might be potent against *M. catarrhalis* and *H. pylori* (Table 1) [21,22]. The therapeutic application of *juglone* is limited by its possible toxicity [113], but this pharmacophore might be used to find more safe and potent inhibitors of **FabZ** and **FabD**.

In Gram-negative bacteria, two enzymes, **FabA** and **FabB**, catalyze the production of UFA [114]. **FabA** has a dual function as a β-hydroxyacyl-ACP dehydratase, catalyzing the dehydration of β-hydroxyacyl-ACP to the *trans*-2-enoyl-ACP (as FabZ does), and as a *trans*-2-,*cis*-3-decenoyl-ACP isomerase, producing the transformation of the *trans*-2-decenoyl-ACP to a *cis*-3-decenoyl-ACP (Figure 1). The size of tunnel in the active site of **FabA**, which perfectly fits *trans*-2-decenoyl-ACP, determines the specificity of the isomerization reaction at the 10-carbon stage of the unbranched substrate [115]. **FabB** specifically elongates the *cis*-UFA produced by **FabA**. **FabA** and **FabI** rival *trans*-2-decenoyl-ACP, and this balance determines the UFA/SFA ratio and the fluidity of the bacterial membrane [116]. *S. pneumoniae* and *Streptococcus mutans* have only one β-hydroxyacyl-ACP dehydrase (**FabZ**), while another enzyme, **FabM**, catalyzes the reaction of double-bond isomerization from *trans*-C_2_-C_3_ to *cis*-C_3_-C_4_ [117,118]. In *E. faecalis*, **FabN** performs the role of **FabA**, and **FabF** elongates the *cis*-UFA produced by **FabN** [119]. **FabQ** from *Aerococcus viridans* can act as a monofunctional dehydrase like **FabZ** or as a bifunctional dehydratase/isomerase like **FabA** (Figure 1) [120]. To produce UFA, some bacteria, particularly *B. subtilis* and *Pseudomonas aeruginosa*, contain fatty acid desaturases, introducing a double bond into saturated acyl chains attached to phospholipids or acyl-CoA [114,121]. *S. aureus* does not contain **FabA** (or its analogs) or any desaturases, but can utilize the exogeneous UFA via acyl-ACP synthetase [36].

Furthermore, *3-decynoyl-N-acetyl cysteamine* is a substrate-mimicking inhibitor of **FabA**, which covalently bonds to the active site of the enzyme [115]. *N42FTA* (3-(pyridin-2-yloxy)aniline and *N*-(4-chlorobenzyl)-3-(2-furyl)-1*H*-1,2,4-triazol-5-amine) may be a promising scaffold to design more potent inhibitors of *P. aeruginosa* **FabA** [122,123].

Many compounds targeting **FabI** (including those undergoing clinical trials) are known: the front-line antituberculosis drug *isoniazid*, the common antiseptic *triclosan* (which demonstrates strong antimalarial activity via *P. falciparum* **FabI** inhibition), *diazaborines*, *CG400462*, *CG400549*, *MUT056399*, *AFN-1252*, *AFN-1720*, *xanthorrhizol*, *benzoxaboroles*, and their derivatives (Table 1) [15,24,26,27,28,124,125,126,127,128,129,130,131,132,133,134]. **FabI** inhibition, in the cases of *triclosan*, *CG400462*, *CG400549*, *MUT056399*, *AFN-1252*, *xanthorrhizol*, and *benzoxaboroles*, was validated by the isolation of resistant clones (*Staphylococci* and *Chlamydia*) containing mutations in the **FabI** gene [28,127,128,131,134,135]. A series of 2*,9-disubstituted 1,2,3,4-tetrahydropyrido [3,4-b]indoles*, *1,4-disubstituted imidazoles*, *1-benzyl-1H-benzimidazoles*, and *4-pyridone* derivatives, and *piperazine* and *imidazole coumarin* derivatives, as well as *N-carboxy pyrrolidine* analogs inhibiting *S. aureus* and/or *E. coli* **FabI**, were also reported [136,137,138,139,140]. Some natural macrocyclic compounds (*complestatin*, *neuroprotectin*, and *chloropeptin*), methyl-branched fatty acids (*14-methyl-9(Z)-pentadecenoic* and *15-methyl-9(Z)-hexadecenoic acids*), *meleagrin*, *phellinstatin*, *chalcomoracin*, and *moracin C* demonstrated a promising ability to target *S. aureus* **FabI** (Table 1) [23,25,29,30]. In addition to the *isoniazid*, the *trans*-2-enoyl-ACP reductase from *M. tuberculosis* (**InhA**), which is involved in the biosynthesis of long-chain fatty acids (LCFA) (mycolic acids), was shown to be a target for several *triclosan* and *benzodiazborine* derivatives; more specific inhibitors of **InhA** were also developed [141,142,143,144,145,146,147,148,149,150]. According to Table 1, *triclosan*- and *complestatin*-related compounds are characterized by similar low IC_50_ values against *S. aureus* **FabI**. Taking into account that *triclosan*’s application is limited by the possibility of bacterial resistance development via different mechanisms, including mutations in the genes of **FabI** or multidrug efflux pump [151,152,153,154] and significant cytotoxic effects [155], the search for natural macrocyclic compounds that are able to inhibit **FabI** seems to be a more promising approach to identify novel antibiotics.

It should be noted that four enoyl-ACP reductase isozymes have been reported in bacteria (Figure 1). *S. pneumoniae, E. faecalis*, and *Clostridia* have **FabK** instead of **FabI**, and **FabV** was discovered in *Vibrio cholerae.* Moreover, some pathogens have more than one enoyl-ACP reductase; for example, **FabI** and **FabK** in *pseudomonads* and *enterococci*, **FabI** and **FabL** in *B. subtilis*, and **FabI**, **FabK**, and **FabV** in *P. aeruginosa* [156,157,158,159]. *Triclosan*, inhibiting **FabI**, is a poor inhibitor of **FabL** and has no activity against **FabK** and **FabV** [157]. **FabMG**, isolated from the soil metagenome, was predicted to be a novel *triclosan*-resistant enoyl-ACP reductase, revealing that the main mechanism to develop *triclosan* resistance is a mutation of **FabI** [160]. *Indole naphthyridinones* and *aquastatin A* are inhibitors of both **FabI** and **FabK** [31,161], while *AG205*, *atromentin*, and *leucomelone* are thought to be specific to **FabK** (Table 1) [32,162]. *Carfilzomib* showed high binding affinity with *Klebsiella pneumoniae* **FabI** and **FabV** [163]. Despite the fact that *atromentin* has a low IC_50_ against *S. pneumoniae* **FabK** (Table 1), compounds that are able to inhibit various enoyl-ACP reductase isozymes (in particular, *complestatin*, *aquastatin A*, and *carfilzomib*) are more preferable for the development of broad-spectrum antibacterials. 

The length of the hydrocarbon chains of the membrane lipids of bacteria is determined by competition between elongating KASs (**FabF/FabB**) and acyltransferases for acyl-ACP produced by *trans*-2-enoyl-ACP reductases (**FabI/FabK/FabL/FabV**). The upper limit is defined by the substrate specificity of the elongating KASs, while the lower limit is a result of the specificity of acyltransferases [164,165,166]. Glycerol-3-phosphate acyltransferases transfer two acyl chains from two acyl-ACPs to the 1 and 2 positions of glycerol-3-phosphate to produce phosphatidic acid (PA), a universal precursor of bacterial phospholipids (Figure 1). Two various acyltransferase systems, **PlsX/PlsY/PlsC** and **PlsB/PlsC**, have been discovered [167,168].

The more widespread bacterial pathway for the formation of PA involves the sequential transfer of acyl from acyl-ACP to acyl-phosphate (acyl-PO_4_) via phosphate acyltransferase (**PlsX**) and then to the 1 position of glycerol-3-phosphate to produce lysophosphatidic acid (LPA) through acyl-phosphate:glycerol-3-phosphate acyltransferase (**PlsY**) [164]. Acyl-PO_4_ is a single substrate for **PlsY**; it cannot utilize acyl-ACP or acyl-CoA [168]. A series of stabilized acyl-phosphate mimetics, including *acyl-phosphonates*, reverse *amide-phosphonates*, and *acyl-sulfamates*, demonstrated promising activity against *S. pneumoniae*, *Bacillus anthracis*, and *S. aureus* through the inhibition of **PlsY** (Table 1) [33,34]. The lead compound, having a low IC_50_ against *S. pneumoniae* **PlsY**, *(Z)-1-oxooctadec-11-enylphosphoramidic acid*, demonstrated potential toxicity [33]. These data necessitate a further search for ways to expand the therapeutic window of acyl-phosphate mimetics. 

A glycerol-3-phosphate acyltransferase (**PlsB**) catalyzes the ligation of the acyl chain to the 1 position of glycerol-3-phosphate to produce LPA. A 1-acyl-sn-glycerol-3-phosphate acyltransferase (**PlsC**) ligates the second acyl chain (in the 2 position) to the LPA produced by **PlsX/PlsY** or **PlsB** to form PA. The advantage of the existence in bacteria of two distinct pathways, the **PlsB/PlsC** and **PlsX/PlsY/PlsC** acyltransferase systems, is the possibility of using exogenous fatty acids, because **PlsB/PlsC** might utilize not only the acyl-ACP produced by *trans*-2-enoyl-ACP reductases (**FabI/FabK/FabL/FabV**) but also the acyl-CoA thioesters derived from exogenous fatty acid metabolism—for example, those produced by **FabD** [168,169]. A fatty acid-rich environment in the host might facilitate the pathogen strain’s resistance to FASII inhibitors by enhancing the assimilation of exogenous fatty acids [170,171]. *S. aureus* can become insensitive to the **FabI** inhibitor *triclosan* via mutations in **FabD**, lowering **FabD** activity and inducing the integration of exogenous fatty acids [171,172]. The UFA-to-SFA ratio, membrane fluidity, and cell growth of *Rhodobacter sphaeroides* were reinstated upon both the inhibition of **FabI** with *diazaborine* and the introduction of exogenous UFA [173].

The cyclopropanation of fatty acids is intended to rigidify the bacterial membranes under stress conditions [174,175,176,177,178]. Cyclopropane fatty acids (CFA) are synthesized by cyclopropane fatty acid acyl-phospholipid synthase (**CfaS**) via the addition of a methylene group to the *cis* double bonds of the UFA chains of membrane phospholipids (Figure 1) [179]. *Dioctylamine* inhibits the **CfaS** of *H. pylori* (Table 1), preventing bacterial insensitivity to acid stress, antibiotics, and macrophage killing [35]. *M. tuberculosis* produces a number of cyclopropanated lipids, including mycolic acids, which are essential components to maintain cell wall integrity (Figure 1). This may indicate that agents capable of inhibiting the lipid cyclopropanation enzymes may be an approach to combatting tuberculosis pathogens [180,181].

#### 2.1.2. Biosynthesis of Head Groups of Bacterial Lipids

**Figure 2:**
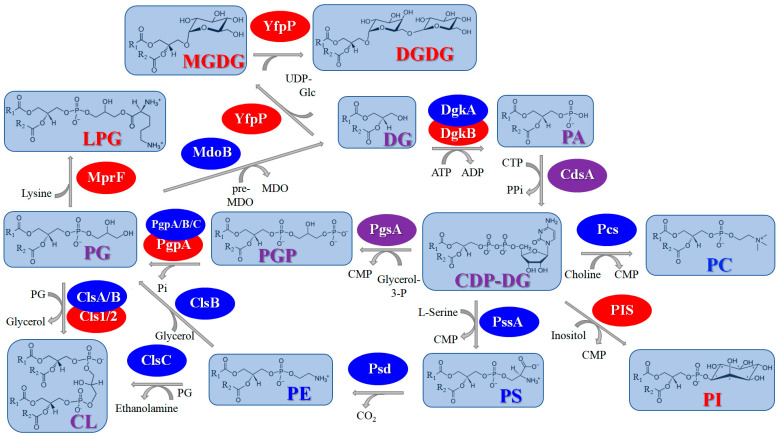
Schematic representation of the synthesis of “heads” of bacterial membrane lipids. The red, blue, and purple ellipses indicate that the enzyme is produced by Gram-positive, Gram-negative, or both Gram-positive and Gram-negative bacteria, respectively. Some examples of enzyme inhibitors are shown in a black box. Abbreviations: **CdsA**—cytidine diphosphate-diacylglycerol synthase; **PgsA**—phosphatidylglycerophosphate synthase; **PgpA**, **PgpB**, and **PgpC**—phosphatidylglycerolphosphate phosphatases; **ClsA**, **ClsB**, **ClsC**, **Cls1**, and **Cls2**—cardiolipin synthases; **PssA**—phosphatidylserine synthase; **Psd**—phosphatidylserine decarboxylase; **Pcs**—phosphatidylcholine synthase; **PIS**—phosphatidylinositol synthase; **MprF**—lysil phosphatidylglycerol synthase and flippase (multiple peptide resistance factor); **MdoB**—phosphoglycerol transferase; **DgkA** and **DgkB**—diacylglycerol kinases; **YpfP**—diacylglycerol β-glucosyltransferase; PA—phosphatidic acid; CDP-DG—CDP-diacylglycerol; PS—phosphatidylserine; PE—phosphatidylethanolamine; PC—phosphatidylcholine; PI—phosphatidylinositol; PGP—phosphatidylglycerol phosphate; PG—phosphatidylglycerol; CL—cardiolipin; LPG—lysil phosphatidylglycerol; DG—diacylglycerol; MGDG—monoglycosyl-DG; DGDG—diglycosyl-DG; R_1_ and R_2_—fatty acid hydrocarbon radicals.

In addition to the enzymes participating in the synthesis of the fatty acids of bacterial membrane lipids, there are unique enzymes that are involved in the synthesis of the heads of lipid molecules. Figure 2 summarizes the information about the synthesis of the lipid heads.

Cytidine disphosphate-diacylglycerol synthase (**CdsA**) is the critical enzyme catalyzing the production of the key intermediate in phospholipid diversity, CDP-diacylglycerol (CDP-DG), from cytidine triphosphate (CTP) and PA (Figure 2). CDP-DG is a precursor of the major phospholipids, including phosphatidylglycerol (PG), cardiolipin (CL), phosphatidylethanolamine (PE) (produced through the decarboxylation of phosphatidylserine (PS)), and even phosphatidylinositol (PI) and phosphatidylcholine (PC), which is absent in most prokaryotic cells. The fundamental nature of the CDP-DG-dependent pathway, characteristic of both prokaryotic and eukaryotic phospholipid biosynthesis [182,183], makes the majority of CDP-DG-converting enzymes poor targets for antibiotic therapy.

Phosphatidylglycerol phosphate (PGP) is synthesized by phosphatidylglycerophosphate synthase (**PgsA**) from CDP-DG via the displacement of cytidine monophosphate (CMP) by glycerol-3-phosphate (glycerol-3-P) (Figure 2). Further, phosphatidylglycerolphosphate phosphatase (**PgpA**) dephosphorylates PGP to yield PG (Figure 2). Two additional genes of phosphatidylglycerolphosphate phosphatases, **PgpB** and **PgpC**, were discovered in *E. coli* [184]. Subsequently, a cardiolipin synthase (**ClsA**) utilizes two PG molecules to produce CL and glycerol (Figure 2). Two extra CL synthases have been discovered in *E. coli*, **ClsB** and **ClsC** [185]. The first one can use one molecule of PG and the molecule of another phospholipid as the second substrate [186]. Moreover, **ClsB** of *E. coli* can convert PE and glycerol into PG in a **PgsA**-independent manner [187]. To form CL, **ClsC** uses PG and PE instead of the two PG molecules [185]. The products of the **Cls1** and **Cls2** genes of *S. aureus* are CL synthases with various types of stress-activated production [188]. Three genes of CL synthases have been identified in *B. subtilis* [189].

The phosphatidylserine synthase (**PssA**) synthesizes PS from CDP-DG via the displacement of CMP by serine (Figure 2). PS is only a minor biosynthetic intermediate in most bacteria and is decarboxylated by phosphatidylserine decarboxylase (**Psd**) to produce PE.

PC is also absent in most prokaryotic cells, although some Gram-negative bacteria contain phosphatidylcholine synthases (**Pcs**) to condense choline into the phosphatidyl moiety of CDP-DG, similar to PssA with serine (Figure 2) [190,191].

One more component that is rarely present in bacterial membranes is PI. For example, *Mycobacteria* are able to form PI using phosphatidylinositol synthase (**PIS**) via the exchange of the CMP moiety of CDP-PG for inositol (Figure 2) [192]. Due to the lack of sequence homology between bacterial and mammalian **PIS**s, their different kinetic characteristics, and the essential role of PI in mycobacteria, the **PIS** of mycobacteria seems to be a good potential drug target for antimycobacterial therapy. Structural analogs of inositol were shown to be more potent inhibitors of mycobacterial **PIS** compared to mammalian **PIS** [193]. Alternatively, there is a difference in the bacterial and mammalian biosynthetic pathways used to form PI: in *Mycobacteria*, PI is produced from CDP-DG and inositol 1-phosphate through an intermediate, phosphatidylinositol phosphate (PIP), which is dephosphorylated subsequently to PI, and inositol 1-phosphate analogs serving as inhibitors of PIP synthase can be used as antimycobacterials [194].

In some Gram-positive bacteria, the anionic glycerophospholipids, particularly PG and CL, can be decorated with aminoacyl residues, most often with lysil, to form cationic PG and CL derivatives by lysil phosphatidylglycerol (LPG) synthase and flippase, multiple peptide resistance factors (**MprF**) (Figure 2). This pathway is crucial for the adaptation of bacteria to cationic antimicrobial peptides [195,196,197]; for this reason, **MprF**-targeting antibodies or inhibitors of the factors involved in **MprF** regulation might sensitize resistant strains to antimicrobial agents [198,199].

In Gram-negative bacteria, a phosphoglycerol transferase (**MdoB**) transfers sn-1-phosphoglycerol from PG to membrane-derived oligosaccharides (MDO) to obtain diacylglycerol (DG). Further, it is phosphorylated by DG kinases (**DgkA** and **DgkB** in Gram-negative and Gram-positive bacteria, respectively) to generate PA, which can be recycled in the phospholipid biosynthetic pathway (Figure 2). **DgkA** presents a large family of prokaryotic DG kinases that are unrelated to the eukaryotic DG kinases and **DgkB** [200]. Some products of the *dgkA* gene are undecaprenol kinases [201].

In Gram-positive bacteria, DG is used to form glycolipids. A diacylglycerol β-glucosyltransferase (**YpfP**) uses uridine diphosphate-glucose (UDP-Glc) to attach one monosaccharide unit to DG to form monoglycosyl-DG (MGDG) and to add one more Glc residue to MGDG to yield diglycosyl-DG (DGDG) (Figure 2). Although **YpfP** is a viable target for the development of novel antibacterial drugs [202], there are no approved inhibitors for this enzyme. Anionic glycopolymers, called lipoteichoic acids, composed of 1,3-polyglycerol-phosphate attached to DGDG (anchoring lipoteichoic acids in the membrane), are exposed on the cell walls of Gram-positive bacteria.

#### 2.1.3. Biosynthesis of Lipid A

**Figure 3:**
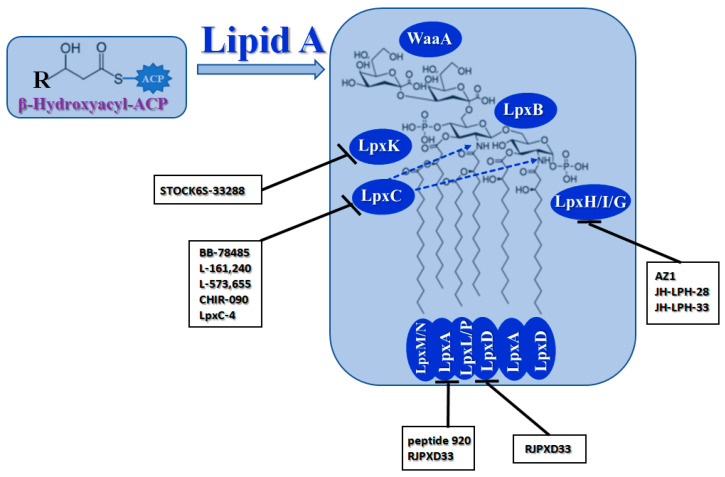
Schematic representation of the biosynthesis of lipid A. The blue ellipses indicate that all enzymes are only produced by Gram-negative bacteria. Some examples of enzyme inhibitors are shown in a black box. Abbreviations: **LpxA**—UDP-N-acetylglucosamine acyltransferase; **LpxC**—UDP-3-*O*-(*R*-3-hydroxyacyl)-*N*-acetylglucosamine deacetylase; **LpxD**—UDP-3-*O*-(*R*-3-hydroxyacyl)glucosamine N-acyltransferase; **LpxH, LpxI**, and **LpxG**—UDP-diacylglucosamine pyrophosphohydrolases; **LpxB**—lipid-A-disaccharide synthase; **LpxK**—tetraacyldisaccharide-1-phosphate 4′-kinase; **WaaA**—3-deoxy-D-*manno*-oct-2-ulosonic acid (Kdo) transferase; **LpxL**, **LpxM**, and **LpxP**—lysophospholipid acyltransferases.

The outer leaflets of the outer membranes of Gram-negative bacteria are formed by specific lipopolysaccharides (LPS). They consist of O-antigen and core sugar domains and a lipid anchor, known as lipid A. This phosphorylated disaccharide lipid is highly conserved and absolutely required for bacterial growth and survival [203,204]. For this reason, many enzymes involved in lipid A biosynthesis have been identified as targets for antibiotic development [205] (Figure 3, Table 2).

A UDP-N-acetylglucosamine acyltransferase (**LpxA**) induces the first step of lipid A biosynthesis (Raetz pathway). It transfers a β-hydroxyacyl chain from β-hydroxyacyl-ACP generated by **FabG** to the 3 position of UDP-N-acetyl-glucosamine (UDP-GlcNAc) (Figure 3). It should be noted that LPS-producing enzymes are highly selective towards ACP thioesters; they cannot be substituted by normal fatty acids [220]. **LpxA** enzymes are highly specific regarding the acyl chain length. For example, *E. coli* **LpxA** transfers only β-hydroxymyristoyl chains [221]. Peptide (*Peptide 920*, *RJPXD33*) and small-molecule inhibitors (particularly *(R)-(3-(2-chloro-6-methoxybenzyl)morpholino)(3-(4-methylpyridin-2-yl)-1H-pyrazol-5-yl)methanone* and *erythroskyrin*) of **LpxA** were reported to compete with the substrate or interact with the complex product (Table 2) [206,207,208,222,223,224,225,226,227]. Analyzing Table 2, one can conclude that *peptide 920* is of interest due to its relatively low IC_50_ value, while *RJPXD33* demonstrates dual targeting of **LpxA** and **LpxD**, offering the possibility to develop novel dual-binding antimicrobials. However, systematic studies of the safety of the peptide’s administration must be performed before it can be determined how promising these methods are. 

The acyl transfer reaction by **LpxA** is thermodynamically reversible and unfavorable, and the subsequent second reaction of the Raetz pathway, catalyzed by UDP-3-*O*-(*R*-3-hydroxyacyl)-*N*-acetylglucosamine deacetylase (**LpxC**), should occur (Figure 3). **LpxC** splits the acetyl radical from the UDP-3-(β-hydroxyacyl)-*N*-acetylglucosamine to produce UDP-3-(β-hydroxyacyl)-D-glucosamine (acyl-UDP-GlcN). Small-molecule inhibitors of the **LpxC** have been discovered, including hydroxamate-based compounds, exemplified by *TU-514*, *BB-78484*, *BB-78485*, *L-159,692*, *L-161,240*, *L-573,655*, *CHIR-090*, *LPC-009*, *LPC-011*, and *LpxC-4* (Table 2) [209,210,211,212,213,214,215,228,229,230,231,232,233,234]. Some of them are highly potent and have proven to be active against various multidrug-resistant Gram-negative bacteria. Analyzing Table 1, it can be assumed that the greatest interest regarding the design of new antibacterials targeting LpxC is in the further optimization of the most effective compounds, *L-161,240*, *CHIR-090*, and *LpxC-4*, in order to avoid emerging resistance [214,234,235,236,237].

A UDP-3-*O*-(*R*-3-hydroxyacyl)glucosamine N-acyltransferase (**LpxD**) performs the third reaction of the lipid A biosynthetic pathway; it transfers a second acyl group from β-hydroxyacyl-ACP to acyl-UDP-GlcN to produce UDP-2,3-bis(β-hydroxyacyl)-D-glucosamine (Figure 3). Some **LpxA** inhibitors, particularly *RJPXD33*, also bind to and inhibit **LpxD** [207,222,224]. It is also believed that **LpxD** is a drug target of natural compounds like *curcumin*, *gallotannin*, *isoorientin*, *neral*, *isovitexin*, *vitexin*, *allicin*, *ajoene*, and *cinnamaldehyde* [237]. Several synthetic compounds related to *hydro-pyrazolo-quinolinones* were identified as **LpxD** inhibitors (Table 2) [216]. 

A UDP-diacylglucosamine pyrophosphohydrolase (**LpxH**) hydrolyses UDP-2,3-bis(β-hydroxyacyl)-D-glucosamine to split UMP and to generate 2,3-diacylglucosamine-1-phosphate (lipid X) (Figure 3). **LpxI** and **LpxG** are functional orthologs of LpxH in α-proteobacteria and in *Chlamydiae*, respectively [238,239,240]. **LpxH** is inhibited by *sulfonyl piperazine* antibiotics (such as *AZ1*, *JH-LPH-28*, *JH-LPH-33*) (Table 2) [217,218,219,241,242,243]. Bacterial efflux pump functioning was found to be a significant deterrent for LpxH-targeting antimicrobials, highlighting the significance of their combination with antibiotics, permeabilizing the outer membrane to fight multidrug-resistant Gram-negative pathogens [218].

A lipid-A-disaccharide synthase (**LpxB**) combines the substrate and the product of the **LpxH**-catalyzed reaction to form the lipid A disaccharide (Figure 3). Compounds that target LpxB have not been discovered to date; only antisense pPNA technology is used to block the *lpxB* gene [244].

A tetraacyldisaccharide-1-phosphate 4′-kinase (**LpxK**) translocates the gamma-phosphate of ATP to the 4′ position of the lipid A disaccharide to produce lipid IV_A_ (Figure 3). The *5-(4-carbamoylbenzenesulfonyl)-N-hydroxy-1H-imidazole-2-carboxamide* analogs (*STOCK6S-33288*, *35740*, *37164*, *39892*, and *43621*) are believed to be a promising template to develop novel potent **LpxK** inhibitors [245].

A 3-deoxy-D-*manno*-oct-2-ulosonic acid (Kdo) transferase (**WaaA**/**KdtA**) adds two Kdo residues to lipid IV_A_ to form Kdo_2_-lipid IV_A_ (Figure 3). Lysophospholipid acyltransferases, **LpxL** and **LpxM**, incorporate two additional acyl chains at positions 2′ and 3′ of Kdo_2_-lipid IV_A_ to yield a hexa-acylated Kdo_2_-lipid A (Figure 3). At lower temperatures, **LpxP** might partially perform the function of **LpxL**. The structure of the active sites of **LpxA** and **LpxD** permits the incorporation of myristoyl residues, while acyltransferases **LpxL**, **LpxP**, and **LpxM** transfer lauroyl, palmitoleoyl, and myristoyl chains, respectively [246,247]. **LpxN** is an ortholog of **LpxM** in *V. cholerae* [248]. No information about the specific compounds inhibiting the enzymes in the last steps of LPS biosynthesis are available in the literature.

### 2.2. Agents with Direct Action on Bacterial Lipid Membranes

**Figure 4:**
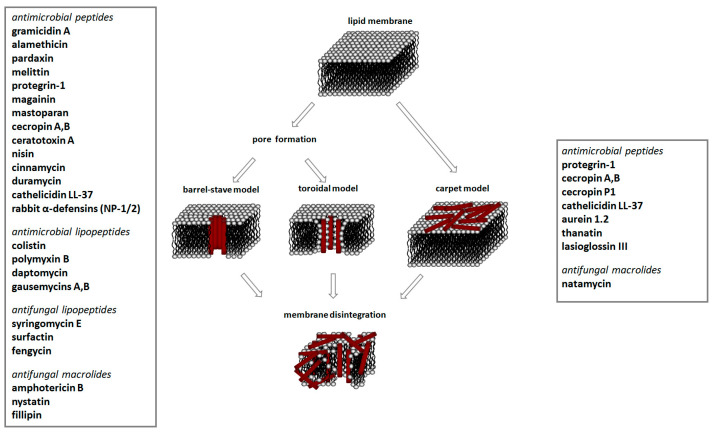
Schematic representation of major mechanisms of antimicrobial action via pore formation (described by two distinct models: barrel-stave channel and toroidal pore-containing lipids) and membrane disruption. Some examples of antibacterials and antifungals directly targeting lipid membranes by two different mechanisms are shown in black boxes.

Figure 4 summarizes the major mechanisms of the direct action of antibacterial agents on target lipid membranes. The mechanisms include pore formation and a detergent-like manner of action [249]. In the first case, the bacterium dies due to a violation in the water–salt balance via the formation of unauthorized transport pathways for water, ions, and small organic molecules. In the second case, the cause of death is the destruction of the membrane after reaching a critical detergent concentration, and a dramatic enhancement in the membrane fluidity and micellization of membrane lipids.

Antimicrobial peptides are synthetized as components of the immune system in higher eukaryotes to defend them against a wide variety of invasive pathogens [250,251]. Antimicrobial lipopeptides are produced in bacteria or fungi as metabolites and/or to gain a competitive advantage over other species. A number of natural antimicrobial agents exert their defending activities primarily via pathogens’ membrane disruption due to pore formation or the disordering of membrane lipids, and they are characterized by a lower probability of inducing microbial resistance. Thus, owing to the high efficiency of these compounds, their broad-spectrum bactericidal effects, and the low rate of pathogens’ resistance to them, the use of antimicrobial peptides and lipopeptides in clinical practice, as well as in the search for new “natural” antibiotics, seems to be a productive anti-infective therapeutic strategy [252,253]. As a rule, antimicrobial peptides and lipopeptides share common structural features, such as molecular amphiphilicity and a net-positive electrical charge, which govern the binding and permeabilization of the negatively charged bacterial membranes through the mechanisms indicated above.

Table 3 presents examples of natural antimicrobial peptides and lipopeptides, their possible lipid targets, and the threshold concentrations needed to form pores and disintegrate lipid bilayers, mimicking the membranes of sensitive bacteria. Most of the antimicrobial peptides—gramicidin A from *Bacillus brevis*; alamethicin produced by the fungus *Trichoderma viride*; pardaxin isolated from secretions of the Red Sea Moses sole; melittin and mastoparan isolated from bee and wasp venom, respectively; protegrin-1 found in porcine leukocytes; magainin found in frog skin; ceratotoxins and cecropins discovered in the accessory gland secretion fluid of the insect *Ceratitis capitata* and the hemolymph of *Hyalophora cecropia*, respectively; nisin from *Streptococcus lactis*; cinnamycin and its close analog duramycin from *Streptomyces* sp.; mammalian defensins; human cathelicidin LL-37 [254,255,256,257,258,259,260,261,262,263,264,265,266,267,268,269,270,271,272,273,274]; lipopeptides; colistins (polymyxins) from *Bacillus polymyxa*; daptomycin from *Streptomyces roseosporus*; and gausemycin from *Streptomyces* sp. [108,275,276,277,278,279]—manifest their action via the pore formation mechanism (Table 3). The pores formed by antimicrobial agents are characterized by their different architectures [280]. For example, alamethicin, pardaxin, and seratotoxin A pores are believed to be “barrels” composed of peptide aggregates [271,272,281,282], while mellitin, magainin, and polymyxin B form (lipo)peptide–lipid toroidal pores [108,270,283,284,285] (Figure 4). Some antimicrobial agents are not shown to form transmembrane pores; they act as detergents by forming a peptide “carpet” on the membrane surface (Figure 4). Such properties are exhibited by cecropin P1, lasioglossin III, and aurein 1.2 (Table 3) [264,286,287,288]. The peptide thanatin disrupts the bacterial outer membrane [289]. In the case of cecropins and protegrins, dual activity was found, including pore formation and detergent-like model action [259,264,290]. In any case, it is likely that when a critical antibiotic concentration is reached, regardless of whether the agent can form pores and in what way, an irreversible change in the rheological properties of the lipid bilayer occurs and it will be destroyed [291,292] (Figure 4).

Despite the lower bacterial resistance to the naturally occurring antibiotics acting on the microbial membranes compared to classical antibiotics, including those inhibiting lipid biosynthesis, antimicrobial peptides and lipopeptides are not a panacea for the emergence of resistance in pathogenic bacteria. One of the evolutionary mechanisms by which to develop pathogenic resistance to cationic antibacterial agents is a reduction in the total negative charge of the cell surface of a microorganism to reduce the initial electrostatic binding. Thus, the resistance of *S. aureus* to defensins and protegrins is determined by the activity of **MprF**, an enzyme that modifies phosphatidylglycerol with L-lysine (1.1.2), which, in turn, leads to a decrease in the surface membrane charge and the repulsion of cationic peptides [299]. *Pseudomonas fluorescens* was proposed to diminish the net anionic charge of the cytoplasmic membrane by reducing the content of anionic phospholipids and increasing the concentration of positively charged ornithine–amide lipids that lead to the resistance to the cationic polymyxin B [300]. According to the literature data, a change in the structure of the LPS of Gram-negative bacteria *E. coli*, *Salmonella enterica*, *Salmonella typhimurium*, *K. pneumoniae*, and *P. aeruginosa* induced by the attachment of *L*-arabinose or phosphatidylethanolamine to the phosphate residues of lipid A leads to the emergence of resistance among these microorganisms to polymyxins due to changes in the membrane surface charge [301,302,303,304,305]. In turn, daptomycin is recommended for application as a therapy against β-lactam-resistant *Streptococcus mitis*. The target of daptomycin is thought to be phosphoglycerol [306]. However, *S. mitis* can rapidly develop resistance to daptomycin via loss-of-function mutations in the gene of **CdsA**, which catalyzes the formation of a common phospholipid precursor, CDP-DG (1.1.2); moreover, daptomicin-resistant strains exhibit the absence of anionic phospholipid membrane microdomains composed of CL and PG [307]. Daptomicin resistance in *E. faecalis* was found to be associated with changes in the genes of cardiolipin synthase, **Cls**, and cyclopropane fatty acid synthase, **CfaS** (1.1.2) [308]. The latter indicates that reducing the level of negatively charged lipids is not the only strategy for resistance development by changing the membrane properties; the fatty acid profile is also of fundamental importance. For example, the development of resistance of *S. aureus* to gausemycin A is accompanied by growth in the ratio between the levels of *anteiso*- and *iso*-BCFA [309]. The membrane fluidity is significantly enhanced when *anteiso* acyl chains replace *iso* acyl chains. In contrast, the resistance of *S. aureus* to daptomycin and *Listeria monocytogenes* to nisin develops with an increase in the percentage of SFA compared to BCFA, which should lead to a decrease in membrane fluidity [310,311]. Thus, alterations in the fatty acid profile and rheological properties of the membrane may be another important factor determining the sensitivity of pathogens to antibiotics. Moreover, whether the fluidity of the membrane should be increased or decreased depends on the architecture of the pores formed by a specific antimicrobial agent.

## 3. Antifungal Agents with Lipid-Related Mechanisms of Action

### 3.1. Inhibition of Biosynthesis of Fungal Cell Membrane Components

Fundamentally, fungal walls are all engineered in a similar way and contain the cell membrane and cell wall [312]. The absence of a cell wall in mammalian cells provides an opportunity for the development of antifungal agents that target the enzymes involved in the biosynthesis of cell wall components in fungi, chitin synthase (Chs) and β-1,3-glucan synthase (Fks) [313,314,315]. The resistance of *Aspergillus fumigatus* and *Candida glabrata* to semisynthetic ehinocandin and caspofungin might arise from not only mutations in the Fks gene but also from alterations in the lipid microenvironment of the enzyme due to an increase in dihydrosphingosine and phytosphingosine content [316,317]. Thus, it should be taken into account that although the cell wall is an essential structure, maintaining the integrity and viability of fungal cells, the fungal lipid membrane serves as both a second barrier and a platform for the functioning of the enzymes that are responsible for the cell wall’s biosynthesis (Chs and Fks) [318,319]. The fungal cell membrane is composed of various glycerophospholipids, sphingolipids, and ergosterol. The latter component is particularly interesting in terms of antifungal targeting, since mammalian cell membranes include another sterol, cholesterol.

#### 3.1.1. Biosynthesis of Fatty Acids of Fungal Membrane Lipids

Recently, a discrepancy between the human and fungal FASI has been discovered [320]. The human FAS encoded by the FASN gene is a type Ib FAS. It consists of one polypeptide chain, including seven domains that assemble into homodimers [321]. Yeast FAS belongs to type Ia FAS and includes a heterododecameric complex composed of six subunits α and six subunits β, which are encoded by the genes Fas1 and Fas2 [322]. It was shown that the deletion of the FAS genes in *Cryptococcus neoformans* significantly reduced the growth and virulence of the fungi [323,324,325]. Thus, the differences in fungal and human FAS [320] can, in *Candida albicans*, potentially be used to target broad-spectrum antifungals towards the products of the Fas1 and Fas2 genes. However, the fungal mutants for the corresponding FAS genes could survive due to the utilization of exogenous fatty acids [326], which might significantly reduce the possibilities of anti-FAS therapy. There have been few fruitful efforts to repurpose antibacterial FAS inhibitors. FAS inhibition in *C. neoformans* with the FASII inhibitor *cerulenin* (1.1) drastically reduced the inhibitory concentration of the inhibitor of ergosterol synthesis, fluconazole (2.4) [323]. *Cerulenin* (but not platensimycin and thiolactomycin) was shown to inhibit *Saccharomyces cerevisiae* FAS [327]. The attempts to inhibit a product of the *OLE1* gene, fatty acid Δ9 desaturase, were more successful in terms of targeting fatty acid biosynthesis in *C. albicans* [328,329].

#### 3.1.2. Biosynthesis of Phospholipid Head Groups

**Figure 5:**
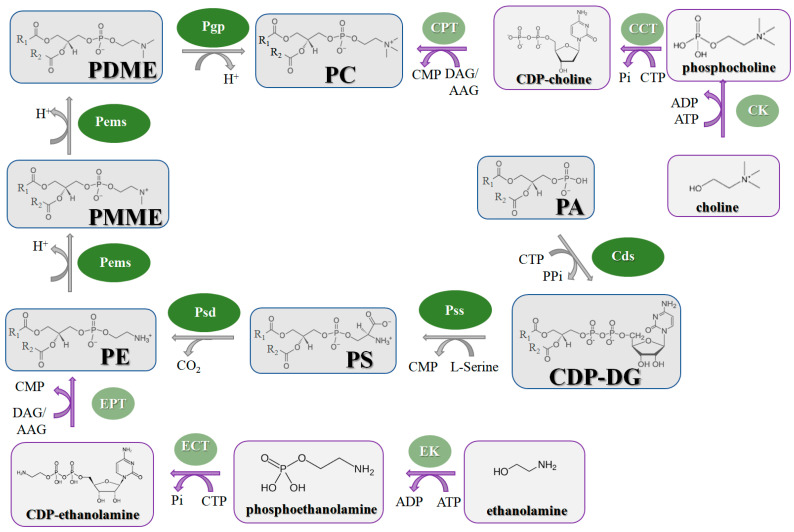
The PC biosynthesis in *S. cerevisiae*. The de novo and Kennedy pathways are represented by the grey and violet lines, respectively. The enzymes of the indicated pathways are highlighted with green and light-green ellipses, respectively. Abbreviations: **Pems**—phosphatidylethanolamine N-methyltransferase; **EK**—ethanolamine kinase; **CK**—choline kinase; **ECT**—phosphoethanolamine cytidylyltransferase; **CCT**—phosphocholine cytidylyltransferase; **EPT**—ethanolaminephosphotransferase; **CPT**—cholinephosphotransferase; PMME—phosphatidyl-N-monomethylethanolamine; PDME—phosphatidyl-*N*,*N*-dimethylethanolamine.

Similar to bacteria (Figure 2), the biosynthesis of the fungal phospholipids begins with the common precursor CDP-DG, which is produced from PA by **CdsA** [330,331]. Further, PI, PGP, and PS are generated from CDP-DG by **PIS**, **PgsA**, and **PssA**, respectively [330,332]. PGP is dephosphorylated by **PgpA** to form PG, and PG is condensed to CL by **ClsA** [330,332]. It is important that the major phospholipid present in most eukaryotic membranes is PC, and PS is a key substrate for PC synthesis in yeast and fungi [333]. The **Psd** enzyme converts PS to PE. The main pathway for PC synthesis in yeast involves the three-step methylation of PE (Figure 5). The first stage includes the methylation of PE by phosphatidylethanolamine N-methyltransferase (**Pems**) to form the phosphatidyl-N-monomethylethanolamine (PMME) and the methylation of PMME to form phosphatidyl-*N*,*N*-dimethylethanolamine (PDME) [330]. PDME is converted to PC by **PgpA**. It was shown that the disruption of PS and PE biosynthesis within the CDP-DG pathway causes the avirulence of *C. albicans* [334]. Moreover, the action of some fungicides is associated with **Pems** inhibition [335]. As, in the cells of higher eukaryotes, PC is mainly synthesized from exogenous ethanolamine and choline via the Kennedy pathway [336,337] (Figure 5), one might suggest that the enzymes that perform the methylation reactions in PC biosynthesis by the CDP-DG pathway can be potential targets for antifungals. However, the alternative Kennedy pathway can be used by lower eukaryotes to produce PC and determines the possibility of developing resistance to the action of such antibiotics.

#### 3.1.3. Biosynthesis of Sphingolipids

**Figure 6:**
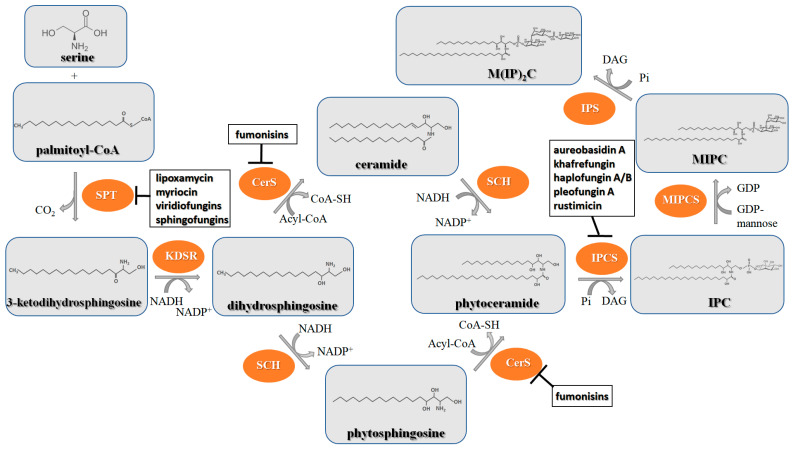
Schematic representation of the sphingolipid biosynthetic pathways in *S. cerevisiae*. The enzymes are highlighted with orange ellipses. Some examples of enzyme inhibitors are shown in black boxes. Abbreviations: **SPT**—serine palmitoyltransferase; **KDSR**—3-ketodihydrosphingosine reductase; **CerS**—ceramide synthase; **SCH**—sphingosine C_4_-hydroxylase; **IPCS**—inositol-phosphoceramide synthase; **MIPCS**—mannosylinositol phosphorylceramide synthase; **IPS**—inositolphosphotransferase; IPC—inositol-phosphoceramide; MIPC—mannose-inositol-phosphoceramide; M(IP)_2_C—mannose-(inositol-P)_2_-ceramide.

Figure 6 demonstrates the pathway for the synthesis of sphingolipids in *S. cerevisiae* [338,339,340]. Serine palmitoyltransferase (**SPT**) performs the condensation of *L*-serine and palmitoyl-CoA to lead to 3-ketodihydrosphingosine [341]. Meanwhile, 3-ketodihydrosphingosine reductase (**KDSR**) converts 3-ketodihydrosphingosine to dihydrosphingosine. Phytoceramide can be synthesized from dihydrosphingosine by ceramide synthase (**CerS**) and sphingosine C_4_-hydroxylase (**SCH**) through two alternative intermediates, ceramide and phytosphingosine (Figure 6). The next reaction involves inositol-phosphoceramide synthase (**IPCS**), which converts phytoceramide to inositolphosphatyl-ceramide (IPC). IPC can be further mannosylated by mannosylinositol phosphorylceramide synthase (**MIPCS**) and condensed with additional inositolphosphate using inositolphosphotransferase (**IPS**) to yield the more complex sphingolipids MIPC and M(IP)_2_C, respectively [341,342,343]. 

Comparing the enzymes in the fungal and mammalian cells required for sphingolipid biosynthesis, it can be concluded that they are homologous and, consequently, are not suitable as targets for the development of low-toxicity antifungal drugs. Nevertheless, potent inhibitors of fungal sphingolipid biosynthesis are described in the literature, although a proper assessment of their possible toxicity has not been completed yet. Mainly, the compounds target **SPT** and **IPCS**. Table 4 summarizes the available information about the agents targeting the synthesis of sphingolipids in different fungi, their chemical structures, and the IC_50_ values against appropriate fungal enzymes. In particular, a variety of different **SPT** inhibitors have been isolated, including lipoxamycin [344,345], myriocin [346], sphingofungins [347,348,349], and viridiofungins [350]. It is interesting that sphingofungins are characterized by similar activity against *C. albicans* and *S. cerevisiae* **SPT**, while *viridiofungins* show 70-200-fold higher selectivity towards *C. albicans* **SPT** (Table 4). Unfortunately, **SPT** inhibitors demonstrated high toxicity towards mammalian cells due to the inhibition of human SPT1 [351]. Interestingly, the *S. cerevisiae* **SPT** is composed of three different subunits, known as Lcb1, Lcb2, and Tsc3, and a homologue of Tsc3 has not been found in mammals [352]. Thus, the question of whether Tsc3 inhibition would be sufficient to effectively suppress the fungal **SPT** activity in pathogenic fungi awaits elucidation. *Fumonisins* are effective inhibitors of **CerS**, but also demonstrate toxicity to mammalian cells [353]. *Australifungin* is a very potent inhibitor of fungal **CerS** from several species; however, the α-diketone and β-ketoaldehyde functional groups present in this compound have high chemical reactivity, which seriously limits *australifungin*’s use [348]. The inositol-phosphoceramide synthase (**IPCS**) identified in *S. cerevisiae* and other different fungi [354] is a potential target for antifungals. **IPCS** inhibitors include *aureobasidin A* [355], *khafrefungin* [356], *haplofungins* [357], and *pleofungin* [358]. *Galbonolide A* and *B* inhibit the **IPCS** of *B. cinerea* and *C. neoformans* [359,360,361]. An analysis of Table 4 shows that *khafrefungin*, *pleofungin A*, and *galbonolide A*, also known as *rustmicin*, are the most potent inhibitors of *C. albicans*, *A. fumigatus*, and *C. neoformans* **IPCS**, respectively. *Khafrefungin* seems to be a more promising candidate due to its relevant selectivity between fungal and mammalian **IPCS** [356,362], while *rustmicin*’s application is limited by its low metabolic stability and drug efflux in fungi [359]. Thus, a further search may accelerate the discovery of selective low-toxicity natural inhibitors for fungi.

#### 3.1.4. Ergosterol Synthesis

**Figure 7:**
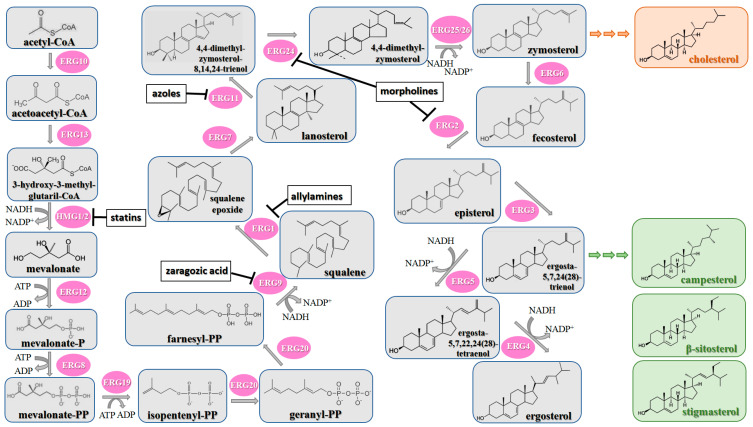
Schematic representation of the ergosterol biosynthetic pathway in *S. cerevisiae*. The enzymes are highlighted with pink ellipses. The branch for the cholesterol biosynthesis in mammalian cells is marked with an orange color. The branch for the biosynthesis of phytosterols (campesterol, β-sitosterol, and stigmasterol) is marked with a green color. Some examples of enzyme inhibitors are shown in the black box. Abbreviations: **ERG10**—acetyl-CoA C-acetyltransferase; **ERG13**—hydroxymethylglutaryl-CoA synthase; **HMG1/2**—hydroxymethylglutaryl-CoA reductase; **ERG12**—mevalonate kinase; **ERG8**—phosphomevalonate kinase; **ERG19**—diphosphomevalonate decarboxylase; **ERG20**—farnesyl diphosphate synthase; **ERG9**—squalene synthase; **ERG1**—squalene epoxidase; **ERG7**—2,3-oxidosqualene cyclase; **ERG11**—lanosterol 14α-demethylase; **ERG24**—sterol C14-reductase; **ERG25/26**—sterol C4-methyloxidase; **ERG6**—sterol C24-methyltransferase; **ERG2**—sterol C8,7-isomerase; **ERG3**—sterol C5(6)-desaturase; **ERG5**—sterol C22-desaturase; **ERG4**—sterol C24-reductase.

Contrary to the cholesterol-containing membranes of mammalian cells, fungal cell membranes are enriched with ergosterol [364]. Figure 7 demonstrates the ergosterol biosynthesis pathway in *S. cerevisiae* [365].

The ergosterol biosynthesis in *S. cerevisiae* includes three different modules, mevalonate, farnesyl-PP, and ergosterol biosynthesis [366]. Table 5 provides the available information about the potential inhibitors of enzymes participating in the ergosterol biosynthetic pathway.

Acetyl-CoA C-acetyltransferase (**ERG10**) catalyzes the additional acetylation of acetyl-CoA molecules to produce acetoacetyl-CoA, which is further transformed by hydroxymethylglutaryl-CoA synthase (**ERG13**) to 3-hydroxy-3-methylglutaryl-CoA. Mevalonate is synthesized by NADPH-dependent hydroxymethylglutaryl-CoA reductase (**HMG1/2**) [366,367]. As the synthesis of mevalonate is the critical step in the ergosterol biosynthetic pathway, it is believed that **HMG1/2** might be a good target for antifungals. It is well known that statins competitively bind to human 3-hydroxy-3-methylglutaryl coenzyme-A reductase, preventing the conversion of 3-hydroxy-3-methylglutaryl-CoA into mevalonate [368,369], and the prospects for the repurposing of statins to treat fungal infections should be estimated. Supporting this theory, *simvastatin*, *lovastatin*, *atorvastatin*, *pravastatin*, *fluvastatin*, and related compounds were reported to decrease the intracellular ergosterol level via the inhibition of **HMG1/2** in *C. glabrata*, *C. albicans*, *Ustilago maydis*, *Trichothecium roseum*, *S. cerevisiae*, *C. neoformans*, *Zygomycetes*, and *Aspergillus* spp. [370,371,372,373,374,375,376,377,378,379,380,381]. Moreover, statin therapy is associated with a reduction in oral *Candida* carriage in hyperlipidemic patients [382]. Statins also reduced mortality due to the diminishing risk of fungal-related complications in patients with diabetes, hematologic malignancies, and COVID-19 [368,383,384,385].

Further, mevalonate is successively phosphorylated to mevalonate-PP by two different kinases, mevalonate kinase (**ERG12**) and phosphomevalonate kinase (**ERG8**). Diphosphomevalonate decarboxylase (**ERG19**) performs the transformation of mevalonate-PP to isopentenyl-PP [386]. Farnesyl-PP is a product of two successive reactions catalyzed by farnesyl diphosphate synthase (**ERG20**) [387].

Using NADPH, squalene synthase (**ERG9**) produces squalene from farnesyl-PP. Natural fungal metabolites, such as *zaragozic acids*, are potent inhibitors of **ERG9 [388]**; however, due to their high toxicity, the compounds failed to reach the clinical trial phase [389].

Squalene epoxidase (**ERG1**) and 2,3-oxidosqualene cyclase (**ERG7**) catalyze the synthesis of squalene epoxide and lanosterol, respectively. It was found that allylamines, *naftifine*, and *terbinafine* are reversible inhibitors of the *Candida* **ERG1** (Table 5) [390,391]. In addition to allylamines, the thiocarbamates *tolciclate* and *tolnaftate* were also shown to be potent inhibitors of **ERG1** (Table 5) [392]. Table 5 clearly demonstrates that all presented allylamines and thiocarbamates are more effective against *T. rubrum* **ERG1** than against *C. albicans* squalene epoxidase. However, the resistance of *Trichophyton* spp. to *terbinafine,* licensed for the treatment of dermatophytic infections, increases dramatically [393,394,395], creating a serious limitation to its further clinical application. Moreover, there is evidence in favor of *terbinafine*-induced hepatotoxicity [396]. The emerging resistance of dermatophytes to *terbinafine* and the moderate activity of both allylamines and thiocarbamates against *C. albicans* show the need for a further search for highly effective **ERG1** inhibitors.

Lanosterol is converted to zymosterol via two intermediates, 4,4-dimethyl-zymosterol-8,14,24-trienol and 4,4-dimethyl-zymosterol, by lanosterol 14α-demethylase (**ERG11**) and sterol C14-reductase (**ERG24**). *Azoles* were identified as effective inhibitors of **ERG11** (Table 5) via selective coordination with heme iron [397,398] and demonstrated striking antifungal activity against a variety of human fungal pathogens [399,400]. Importantly, *azoles* inhibit human lanosterol 14α-demethylase at substantially higher concentrations than the fungal enzyme [397]. A series of *steroidal 1,4-dihydropyridines* also showed promising activity against various *Candida* spp. via the inhibition of **ERG11** [401]. Despite the pronounced activity of azoles against *Candida* spp. **ERG11** (Table 5), the enlarged resistance to azoles by *Candida* species is a serious threat in their clinical use [402,403]. Therefore, the novel azole-based derivatives could attract attention as **ERG11** inhibitors. 

In the fungal cell, sterol C24-methyltransferase (**ERG6**) transforms zymosterol to fecosterol. It is converted to episterol by sterol C8,7-isomerase (**ERG2**). Sterol C5(6)-desaturase (**ERG3**) converts episterol to ergosta-5,7,24(28)-trienol [404,405,406]. NADPH-dependent sterol C22-desaturase (**ERG5**) catalyzes the formation of the next intermediate, ergosta-5,7,22,24(28)-tetraenol. At the final step, NADPH-dependent sterol C24-reductase (**ERG4**) converts ergosta-5,7,22,24(28)-tetraenol to ergosterol molecules [365]. It was demonstrated that *amorolfine*, *fenpropidin*, *fenpropimorph*, and the related *morpholines* and *piperidines* act as dual inhibitors of **ERG24** and **ERG2** [407,408]. Thus, these compounds seem to be ideal antifungals, as acquiring resistance against them will be difficult for pathogens because of the requirement to mutate two enzyme genes at once. Among the aminopiperidine derivatives, as presented in Table 5, *compound 1b* has a lower IC_50_ value, and the substantial prolongation of survival in infected mice with its oral administration [409] indicates the potential clinical benefits. However, presently, only amorolfine is used clinically to treat nail infections.

It should be noted that zymosterol is a precursor in cholesterol biosynthesis in mammalian cells [410] (this branch of the biosynthetic pathway is marked with an orange color in Figure 7). Ergosta-5,7,24(28)-trienol is a precursor of phytosterols, campesterol, β-sitosterol, and stigmasterol [411] (this branch of the biosynthetic pathway is indicated with a green color in Figure 7).

**Table 5:** Major inhibitors of fungal sterol biosynthesis.

Inhibitor	Structure	Enzyme		IC_50_, μM	References
*terbinafine*	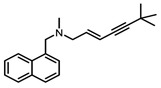	**ERG1**	*C. albicans*	0.03	[390,392]
			*C. parapsilosis*	0.02–0.04	[390]
			*C. glabrata*	0.137	[390]
			*Trichophyton rubrum*	0.002–0.016	[390,392]
			*A. fumigatus*	0.24	[390]
*naftifine*	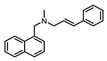	**ERG1**	*C. albicans*	1.1	[390]
			*C. parapsilosis*	0.34	[390]
			*T. rubrum*	0.115 ± 0.030	[392]
*SDZ 87-469*	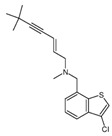	**ERG1**	*T. rubrum*	0.020 ± 0.005	[392]
			*C. albicans*	0.011	[392]
*tolciclate*	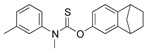	**ERG1**	*T. rubrum*	0.028 ± 0.003	[392]
			*C. albicans*	0.12	[392]
*tolnaftate*	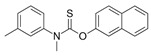	**ERG1**	*T. rubrum*	0.052± 0.009	[392]
			*C. albicans*	1.04	[392]
*bifonazole*	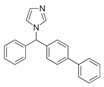	**ERG11**	*C. albicans*	0.3	[397]
*clotrimazole*	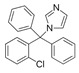	**ERG11**	*C. albicans*	0.091	[397]
*miconazole*	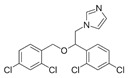	**ERG11**	*C. albicans*	0.072	[397]
*fluconazole*	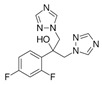	**ERG11**	*C. albicans*	0.051–0.6	[397,412]
			*C. neoformans*	0.17	[413]
			*Malassezia globosa*	0.206 ± 0.008	[414]
*itraconazole*	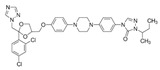	**ERG11**	*C. albicans*	0.039–0.4	[397,412]
			*C. neoformans*	0.17	[413]
			*M. globosa*	0.188 ± 0.008	[414]
*voriconazole*	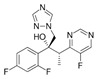	**ERG11**	*C. neoformans*	0.17	[413]
*VT-1129*	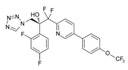	**ERG11**	*C. neoformans*	0.16	[413]
*ketoconazole*	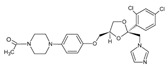	**ERG11**	*C. albicans*	0.064–0.5	[397,412]
			*M. globosa*	0.176 ± 0.016	[414]
*ketaminazole*	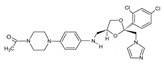	**ERG11**	*M. globosa*	0.321 ± 0.042	[414]
*compound 1a*	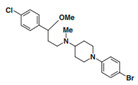	**ERG24**	*C. albicans*	0.063	[415]
*compound 1b*	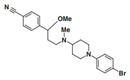	**ERG24**	*C. albicans*	0.016	[415]

### 3.2. Agents with Direct Action on Fungal Lipid Membrane

The principles of action of naturally occurring antibiotics on fungal membranes are similar to those of antibacterial peptides and lipopeptides (Figure 4).

Table 6 summarizes the data concerning the effect of antifungal lipopeptides and polyene macrolides on the permeability of lipid bilayers that mimic the cell membranes of target fungi. One of the most attractive groups is the cyclic lipopeptides, which are secondary metabolites of certain bacteria and are used to combat plant fungal pathogens. It is well known that the syringomycins and syringopeptines from *Pseudomonas syringae*, and surfactins, fengycins, iturins, bacillomycins, and mycosubtilin from *B. subtilis*, form the transmembrane pores in model lipid membranes [416,417,418,419,420,421,422,423,424,425,426,427].

Another clinically important group is antifungal macrolides and polyene antibiotics. Amphotericin B, nystatin, and fillipin demonstrate antimicrobial activity via the formation of transmembrane pores in the target-sterol-containing membranes [432,433,434,438,439,440,441,442,443]. The three-dimensional structure of the amphotericin B channel was proposed as an asymmetric heptameric complex of polyene and sterol molecules penetrating the membrane [444]. In addition to natamycin’s inhibitory effect on transport proteins, it was suggested to specifically interact with the sterol- and sphingomyelin-enriched ordered phase and disrupt lipid packing [437,445]. Amphidinol 3, an antifungal polyene isolated from a marine dinoflagellate, is also able to induce pore-like defects in model membranes [446].

Antimicrobial peptides also demonstrate antifungal efficiency. Piscidins identified in the mast cells of fish exert their fungicidal effects on *C. albicans* by disrupting the fungal membranes through pore formation [436,447]. An antimicrobial peptide from the tree frog *Hyla punctata*, hylaseptin P1-NH_2_, demonstrates strong antifungal potential by promoting membrane disruption [448].

*Saccharomyces cerevisiae* strains, resistant to syringomycin E, are characterized by a decrease in the length of fatty acid chains and sphingolipid content. Mutants were defective in two key enzymes of the terminal sphingolipid biosynthetic pathway, **IPCS** and **IPS** (2.1.3) [449]. The sensitivity of *S. cerevisiae* towards syringomycin E was also shown to depend on the C_4_-hydroxylation of sphingoid bases to form phytoceramide, catalyzed by **SCH** [450]. The relevance of M(IP)_2_C [451] and sphingolipid C_4_-hydroxylation [452] for the lateral segregation of lipids in *S. cerevisiae* membranes might suggest that syringomycin E may interact with sphingolipid-enriched microdomains, and its pore-forming ability is sensitive to their composition [453,454].

As expected for the sterol-dependent mechanism of pore formation by polyene antibiotics, the decline in the ergosterol content in the plasma membranes of target fungi results in the development of resistance [455]. In fact, it was found that the minimal inhibitory concentration of amphotericin B against *C. albicans* was increased by the deletion of **ERG2**, **ERG6**, **ERG3**, and **ERG11**, the enzymes participating in the ergosterol biosynthetic pathway (2.1.4). It should be noted that the emergence of resistance to amphotericin B through a decrease in ergosterol content makes resistant strains extremely sensitive to osmotic and other types of stress. The reduced level of ergosterol in clinical strains of *Candida lusitaniae*, which is resistant to amphotericin B, might arise from mutations in the **ERG3** gene [456].

## 4. Antivirals Targeting Lipid Envelope

Since we have narrowed our focus to reviewing only compounds that directly target pathogen membranes, antivirals that have been shown to affect the membranes of virions, which lead to the destruction of the lipid envelope or suppression of virus fusion with the host cell, are discussed below.

Many socially significant viruses are enveloped, i.e., the virions are surrounded by a supercapsid composed of a lipid bilayer. Despite the fact that the origin of the lipid envelope is the host cell membrane, in some cases, a quantitative difference has been found in the content of various lipids in the viral envelope and the host cell membrane from which the virions have been budded [457,458,459,460,461]. This may also be due to virus-induced changes in the host cell’s lipid metabolism [462,463]. Thus, the lipid membranes of enveloped viruses might be considered a target for innovative antiviral drugs. The compounds are thought to break the lipid envelopes of virions or dramatically change the properties of the viral membrane in order to prevent fusion with the cell membrane. A significant advantage of using such an approach is the broadening of the spectrum of antiviral activity and a decrease in the resistance to viral pathogens.

### 4.1. Disrupting Agents

#### 4.1.1. Photosensitizing Antivirals

**Figure 8:**
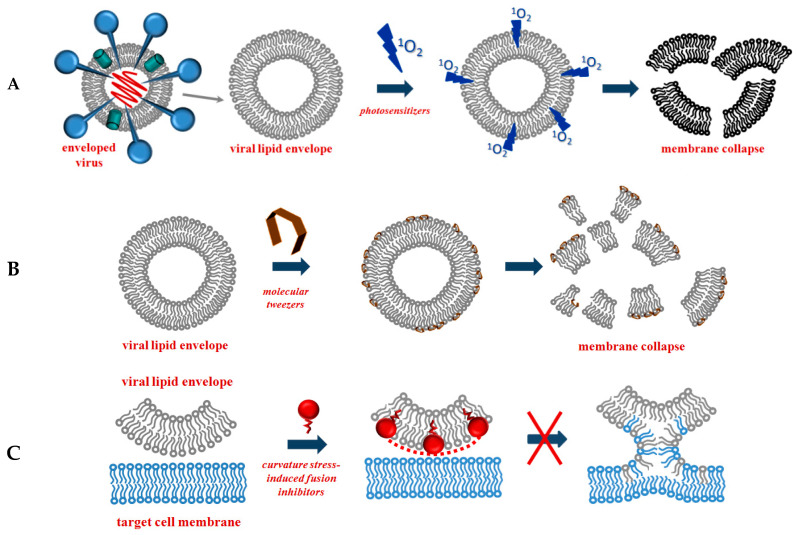
Modes of action of lipid-targeting antivirals: photosensitizers (**A**); molecular tweezers (**B**); curvature stress-induced fusion inhibitors (**C**).

Photosensitizers are compounds that can absorb light and generate reactive oxygen species, which, in turn, leads to the peroxidation of membrane lipids and damage to the lipid bilayers of both viral and cellular membranes [464] (Figure 8A). In the absence of virus systems for reparation, the photodamage of the lipid envelope causes a dramatic reduction in the infectivity of virions due to the inactivation of viral fusion. Among the photosensitizers, compounds with absorption in the infrared region are of particular interest in the search for new broad-spectrum antivirals due to their substantially higher tissue transparency for the radiation of this spectrum [465].

*Hypericin*, a plant-occurring polycyclic quinone, demonstrated broad-spectrum activity against enveloped viruses such as human immunodeficiency virus type 1 (HIV-1), Moloney murine leukemia virus, equine infectious anemia virus, vesicular stomatitis virus (VSV), herpes simplex virus types 1 (HSV-1) and 2 (HSV-2), parainfluenza virus (PIV), vaccinia virus, murine cytomegalovirus (mCMV), and Sindbis virus (SINV) [466,467,468,469,470,471] (Table 7). *Hypericin* did not alter non-enveloped viruses [467]. Halogen derivatives of hypericin were shown to be effective against HSV-1 [472,473]. *Gymnochromes* isolated from *Gymnocrinus richer* were shown to be highly potent antiviral agents against dengue virus, HSV-1, and influenza virus type A (IVA) [474,475] (Table 7). *Hypocrellins* from *Hypocrella bambuase* also demonstrated light-dependent anti-HIV, anti-mCMV, anti-HSV-1, anti-VSV, and anti-IVA efficacy [469,476,477,478] (Table 7), while the non-enveloped virus was not inactivated [477].

Initially, *rigid amphipathic perylene-containing nucleoside derivatives*, particularly 5- (perylen-3-yl)ethynyl-2′-deoxy-uridine (*dUY11*) and 5-(perylen-3-yl)ethynyl-arabino-uridine (aUY11), having considerable activity against IVA, hepatitis C (HCV), HSV-1, HSV-2, mCMV, VSV, SINV, tick-borne encephalitis virus (TBEV), yellow fever virus (YFV), Chikungunya virus (CHIKV), African swine fever virus, PIV, and human respiratory syncytial virus (RSV) (Table 7), were believed to inhibit viral fusion by affecting membrane curvature stress (Section 3.2) [479,480,481]; however, later, their action through the photosensitization of viruses was postulated [482,483,484,485,486,487]. No activity or the non-specific activity of perylene derivatives against non-enveloped viruses was found [480,488]. Non-nucleoside perylene derivatives also showed promising antiviral activity [489].

BODIPY-based photosensitizer 2,6-diiodo-1,3,5,7-tetramethyl-8-(N-methyl-4-pyridyl)-4,4′-difluoroboradiazaindacene (*DIMPy-BODIPY*) exhibited the photodynamic inactivation of dengue virus and VSV at nanomolar concentrations [490].

A class of thiazolidine-based lipophilic inhibitors of *LJ* and *JL* series demonstrating high activity against a variety of enveloped viruses (Table 7), with no effect on the infection of non-enveloped viruses, was also originally described as curvature-induced antivirals (Section 3.2), but it was later shown that the compounds act as membrane-targeted photosensitizers [491,492,493].

Cationic *imidazolyl* and *pyridyl porphyrins* were characterized as promising photosensitizing antivirals against SARS-CoV-2 and HSV-1 [494,495]. A natural *chlorine* photosensitizer, pheophorbide a, inactivates HSV-1, HSV-2, MERS-CoV, SARS-CoV-2, YFV, HCV, and SINV, by targeting the lipid envelope [496,497] (Table 7).

**Table 7:** Photosensitizers and their antiviral activity.

Photosensitizer	Structure	Virus	IC_50_, µM	Reference
*hypericin*	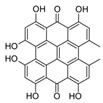	HIV-1	0.44	[466]
		HSV-1	0.006	[469]
*gymnochrome B*	* 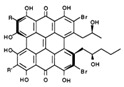 *	dengue	0.029	[475]
*hypocrellin A*	* 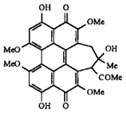 *	HSV-1	0.015	[469]
*hypocrellin B*	* 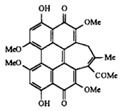 *	HSV-1	0.025	[469]
*5-(perylen-3-yl)ethynyl-2′-deoxy-uridine (dUY11)*	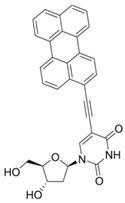	IVA	0.097–2.7	[480,498]
		HSV-1	0.048–0.131	[479]
		HSV-2	0.031–0.055	[479,480]
		HCV	0.183–0.187	[479,480]
		mCMV	0.037 ± 0.016	[480]
		SINV	0.006 ± 0.001	[480]
		TBEV	0.024 ± 0.013	[483,499]
		PIV	2.2 ± 0.5	[498]
		RSV	1.8 ± 0.2	[498]
		SARS-CoV-2	0.2564	[487]
*5-(perylen-3-yl)ethynyl-arabino-uridine (aUY11)*	* 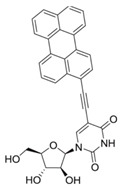 *	IVA	0.078–5.2	[480,498]
		HSV-1	0.048 ± 0.012	[479]
		HSV-2	0.052 ± 0.003	[480]
		HCV	0.107 ± 0.041	[480]
		mCMV	0.013 ± 0.004	[480]
		SINV	0.011 ± 0.005	[479]
		TBEV	0.018 ± 0.010	[483]
		YFV	0.0086 ± 0.0007	[484]
		CHIKV	<0.78	[484]
		PIV	1.3 ± 0.3	[498]
		RSV	2.3 ± 0.1	[498]
		SARS-CoV-2	0.4058	[487]
*(5Z)-5-[(5-phenylfuran-2-yl)methylidene]-3-prop-2-enyl-2-sulfanylidene-1,3-thiazolidin-4-one (LJ-001)*	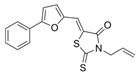	HIV	0.133	[492]
		Newcastle disease virus	0.095	[492]
		Ebola virus	0.9	[492]
		IVA	0.026	[492]
		Nipah virus	0.048	[492]
		Hendra virus	0.018	[492]
		Rift valley fever virus	0.02	[492]
		Semliki forest virus	0.537	[492]
		HSV-1	0.02	[492]
		hCMV	0.13	[492]
		VSV	0.298	[492]
*(Z) 3-ethyl-5-[5-(2-methoxyphenyl)-furan-2-ylmethylene]oxazolid* *-ine-2,4-dithione (JL-103)*	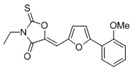	HIV	0.013	[492]
		Newcastle disease virus	0.004	[492]
		Ebola virus	0.185	[492]
		IVA	0.002	[492]
		Nipah virus	0.004	[492]
		Hendra viru	0.0005	[492]
		Rift valley fever virus	0.003	[492]
		Semliki forest virus	0.044	[492]
		HSV-1	0.002	[492]
		hCMV	0.004	[492]
		VSV	0.011	[492]
*5,15-bis(1,3-dimethylimidazol-2-yl)chlorin (ICH-Me2+)*	* 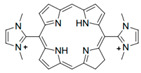 *	SARS-CoV-2	0.12	[494]
*pheophorbide a*	* 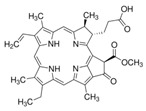 *	SARS-CoV-2	0.18	[497]
		MERS-CoV	0.18	[497]

IC_50_ is determined at photoactivation.

#### 4.1.2. Tweezers

Molecular tweezers are membrane-destabilizing agents that can disrupt the virus lipid envelope and can be used as broad-spectrum antivirals against influenza A virus, respiratory syncytial virus, human immunodeficiency virus, herpes simplex viruses, human cytomegalovirus, Ebola and Marburg viruses, SARS-CoV, SARS-CoV-2, MERS-CoV, and other enveloped viruses. These small molecules act as pincers that bind lipid head groups and disrupt lipid ordering and packing in the virus lipid envelope, which results in the virions being unable to infect the cells [500] (Figure 8B).

A basing compound, *CLR01*, was shown to inhibit HIV-1, Ebola, Zika, herpes simplex (HSV-1, HSV-2), measles, influenza virus, and SARS-CoV-2 infection by directly targeting the viral membrane [501,502,503] (Table 8). Its close analog, *CLR05*, also possessed broad-spectrum antiviral activity [500]. *CLR01* and *CLR05* did not reduce infection by the non-enveloped adenovirus and encephalomyocarditis virus [500]. The membrane-disrupting and, consequently, the antiviral activity of *CLR01* might be substantially potentiated by the introduction of C_4_, C_7_, or aromatic radicals to each phosphate group [500,503] (Table 8).

#### 4.1.3. Antimicrobial Peptides

Although many antimicrobial peptides, especially *defensins* and *cathelicidins*, have been shown to possess antiviral effects [273,504,505], the mechanisms of antiviral action are highly pleiotropic and involve more than simply a direct effect on the viral membrane. Regarding the focus of this section, *bomidin*, a naturally occurring antimicrobial peptide that is active against a variety of enveloped viruses, including SARS-CoV-2, HSV, dengue virus, and CHIKV, was supposed to disrupt the viral membrane [506]. *Plantaricin NC8 αβ*, a two-peptide bacteriocin produced by *Lactobacillus plantarum* strains, was shown to inhibit SARS-CoV-2, IVA, flaviviruses Langat and Kunjin, and HIV-1, via permeabilizing and destroying their envelopes [507] (Table 9). It was demonstrated that the anti-HIV activity of a cyclic peptide from plants, *kalata B1*, resulted from the disruption of the membranes of HIV particles due to their raft-like lipid density and enrichment with PE [508].

### 4.2. Fusion Inhibitors Affecting Membrane Fluidity and/or Curvature Stress

An essential step in the fusion of an enveloped virus with a cell is the fusion of their lipid membranes. It is believed that this occurs in several successive stages, one of which includes the assembly of the contiguous outer lipid leaflets of the membranes to constitute an intermediate stalk. The stalk is characterized by a negative spontaneous curvature, corresponding to this formation via the cone-shaped lipids of an inverted hexagonal phase (H_II_) [509,510]. The induction of positive curvature stress by putative antiviral agents is believed to prevent the generation of fusion intermediates of negative curvature (Figure 8C).

Lipophosphoglycan dramatically reduced the fusion of Sendai virus and IVA with host cells [511,512], while it raised the bilayer-to-H_II_-phase transition temperature of phosphatidyletanolamine, indicating the elevation of positive curvature stress by lipophosphoglycan [511].

Naturally occurring and synthetic lipopeptides appear to be the most promising candidates when taking into account their amphiphilicity and cone shape, which suggests the induction of positive curvature when incorporated into a lipid monolayer. A simple lipopeptide sequence, myr-WD, was shown to successfully combat IVA and murine coronavirus infections by modulating the membrane lipid packing and surface potential [513]. *Surfactin*, a cyclic lipopeptide from *B. subtilis*, was found to inhibit porcine epidemic diarrhea virus and transmissible gastroenteritis virus infections via affecting curvature stress [514]. The dependence of the efficiency of surfactins to inhibit VSV, HSV-1, and Semliki forest virus on the length of the hydrocarbon “tail” was in good agreement with their membrane targeting [515]. Recently, the ability of several lipopeptides to successfully inhibit SARS-CoV-2 fusion with *Vero* cells was shown [516] (Table 10). The most effective compounds were also characterized by their marked ability to increase the transition temperature of phosphatidylethanolamine from the lamellar to H_II_ phase, i.e., induce a positive curvature stress [516]. Interestingly, simpler molecules, such as black pepper alkaloid piperine, show a similar ability to suppress SARS-CoV-2 infection [517].

## 5. Conclusions

(i) Due to principal differences in the organization of fatty acid synthase systems in bacteria and mammals, the specific inhibitors of bacterial key enzymes, especially the acetyl-CoA-carboxylase complex, various β-ketoacyl-ACP synthases, different NADPH-dependent reductases, β-hydroxyacyl-ACP dehydrases, and acyl-phosphate:glycerol-3-phosphate acyltransferase, are attractive targets for the development of low-toxicity antibacterials.
(ii) The pathway for the synthesis of the lipid fatty acid tails in fungi is similar to that in mammalian cells and, therefore, is not very promising in the search for potential antifungals.
(iii) The presence of a single fundamental pathway for the synthesis of the phospholipid heads in both prokaryotes and eukaryotes makes the majority of the involved enzymes poor targets for antibiotic therapy in bacterial and fungal infections.
(iv) Many enzymes of the lipopolysaccharide (Kdo_2_-lipid A) biosynthetic pathway in Gram-negative bacteria (UDP-N-acetylglucosamine acyltransferase, UDP-3-*O*-(*R*-3-hydroxyacyl)glucosamine N-acyltransferase, UDP-3-*O*-(*R*-3-hydroxyacyl)-*N*-acetylglucosamine deacetylase, and UDP-diacylglucosamine pyrophosphohydrolase) are identified as targets for antibiotic development.
(v) Sphingolipid biosynthetic pathways are conserved from yeast to humans, and the enzymes cannot serve as targets for low-toxicity antifungals. Some inhibitors of inositol-phosphoceramide synthase demonstrate promisingly low effective concentrations.
(vi) The most effective approach when targeting fungal lipid biosynthesis is to search for inhibitors of enzymes in the ergosterol pathway, especially squalene epoxidase, lanosterol 14α-demethylase, and sterol C14-reductase/sterol C8,7-isomerase.
(vii) A preference given to inhibitors that simultaneously act on two enzymes of the lipid biosynthetic pathway or the combination of inhibitors with agents directly affecting the pathogen membrane should reduce the risk of developing antibiotic resistance in pathogenic strains.
(viii) Natural antimicrobial agents exert their defensive activities via pathogen membrane disruption due to pore formation or the disordering of membrane lipids. Due to the high efficiency of naturally occurring antimicrobial agents, their broad-spectrum antibacterial/antifungal/antiviral effect, and their low rate of resistance in pathogen strains, the use of antimicrobial peptides, lipopeptides, and polyenes is a good anti-infective therapeutic strategy.
(ix) The lipid envelope of viruses should be considered as a target for innovative antivirals, disrupting the membranes of virions or inducing curvature stress and inhibiting viral entry.

## Figures and Tables

**Table 1:** Major inhibitors of bacterial FASII.

Inhibitor	Structure	Enzyme	Origin	IC_50_, μM	References
*amino-oxazole dibenzylamide*	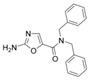	**AccC**	*E. coli*	0.125	[3]
*(R)-2-(2-chlorobenzylamino)-1-(2,3-dihydro-1H-inden-1-yl)-1H-imidazo[4,5-b]pyridine-5-carboxamide*	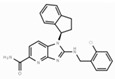	**AccC**	*E. coli*	0.02	[4]
*moiramide B*	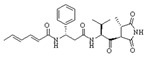	**AccAD**	*S. aureus*	0.096	[5]
			*E. coli*	0.006	[5]
*andrimid*	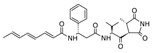	**AccAD**	*S. aureus*	0.091	[5]
			*E. coli*	0.004	[5]
*thiolactomycin*	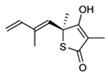	**FabH**	*S. pneumoniae*	7.9 ± 1.1	[6]
			*H. influenzae*	5.8 ± 1.6	[6]
			*M. tuberculosis*	24	[7]
			*E. coli*	32–110	[6,8]
		**FabF**	*E. coli*	6	[8]
		**FabB**	*E. coli*	2–25	[8,9]
*SB418011*	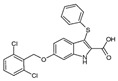	**FabH**	*S. pneumoniae*	0.016 ± 0.003	[6]
			*H. influenzae*	0.59 ± 0.05	[6]
			*E. coli*	1.20 ± 0.40	[6]
*cerulenin*	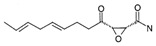	**FabF**	*E. coli*	20	[8]
		**FabB**	*E. coli*	3	[8]
*platensimycin*	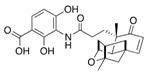	**FabF**	*S. aureus*	0.02–0.29	[10,11]
			*E. coli*	0.02	[12]
*platencin*	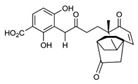	**FabH**	*S. aureus*	9.2–16.2	[10,11]
		**FabF**	*S. aureus*	0.1–4.6	[10,11]
*(-)-epigallocatechin gallate*	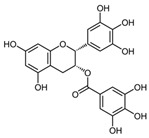	**FabG**	*E. coli*	5	[13]
			*P. falciparum*	0.3	[14,15]
		**FabI**	*E. coli*	15	[13]
			*P. falciparum*	0.2	[14]
		**FabZ**	*P. falciparum*	0.03–0.4	[14,15]
*(-)-gallocatechin gallate*	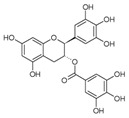	**FabG**	*E. coli*	10	[13]
			*P. falciparum*	1.1	[14]
		**FabI**	*E. coli*	5	[13]
			*P. falciparum*	0.5	[14]
		**FabZ**	*P. falciparum*	0.6	[14]
*(-)-epicatechin gallate*	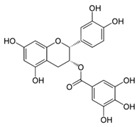	**FabG**	*E. coli*	15	[13]
			*P. falciparum*	1	[14]
		**FabI**	*E. coli*	10	[13]
			*P. falciparum*	0.2	[14]
		**FabZ**	*P. falciparum*	0.4	[14]
*(-)-catechin gallate*	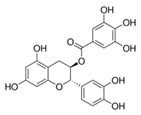	**FabG**	*E. coli*	10	[13]
			*P. falciparum*	1	[14]
		**FabI**	*E. coli*	5	[13]
			*P. falciparum*	0.3	[14]
		**FabZ**	*P. falciparum*	0.4	[14]
*butein*	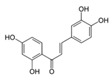	**FabG**	*E. coli*	10	[13]
		**FabI**	*E. coli*	30	[13]
*isoliquiritigenin*	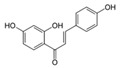	**FabG**	*E. coli*	20	[13]
		**FabI**	*E. coli*	40	[13]
*2,2′,4′-trihydroxychalcone*	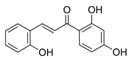	**FabG**	*E. coli*	25	[13]
		**FabI**	*E. coli*	40	[13]
*fisetin*	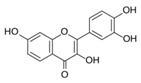	**FabG**	*E. coli*	30	[13]
			*P. falciparum*	4.1	[14]
		**FabI**	*E. coli*	50	[13]
			*P. falciparum*	1	[14]
		**FabZ**	*P. falciparum*	2	[14]
*quercetin*	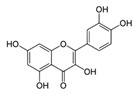	**FabG**	*E. coli*	20	[13]
			*P. falciparum*	5.4	[14]
		**FabI**	*E. coli*	20	[13]
			*P. falciparum*	1.5	[14]
		**FabZ**	*P. falciparum*	1.5	[14]
*resveratrol*	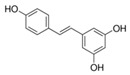	**FabG**	*E. coli*	65	[13]
		**FabI**	*E. coli*	30	[13]
*piceatannol*	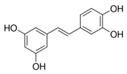	**FabG**	*E. coli*	35	[13]
		**FabI**	*E. coli*	15	[13]
*fustin*	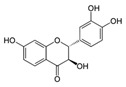	**FabG**	*E. coli*	25	[13]
		**FabI**	*E. coli*	40	[13]
*taxifolin*	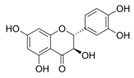	**FabG**	*E. coli*	20	[13]
		**FabI**	*E. coli*	30	[13]
*kaempferol*	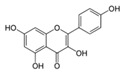	**FabG**	*P. falciparum*	4	[14]
		**FabI**	*P. falciparum*	20	[14]
*luteolin*	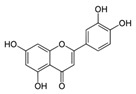	**FabG**	*P. falciparum*	4	[14]
		**FabI**	*P. falciparum*	2	[14]
		**FabZ**	*P. falciparum*	5	[14]
*luteolin 7-O-β-D-glucopyranoside*	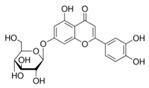	**FabI**	*P. falciparum*	22	[16]
*myricetin*	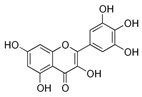	**FabG**	*P. falciparum*	14	[14]
		**FabI**	*P. falciparum*	0.4	[14]
		**FabZ**	*P. falciparum*	2	[14]
*isorhamnetin*	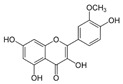	**FabG**	*P. falciparum*	8.3	[14]
		**FabI**	*P. falciparum*	5	[14]
*7,3′,4′-trihydroxyisoflavone*	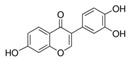	**FabG**	*E. coli*	35	[13]
		**FabI**	*E. coli*	25	[13]
*morin*	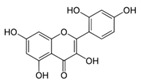	**FabG**	*P. falciparum*	2.3	[14]
		**FabI**	*P. falciparum*	5	[14]
		**FabZ**	*P. falciparum*	8	[14]
*macrolactin S*	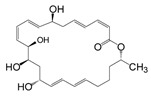	**FabG**	*S. aureus*	130	[17]
*macrolactin B*	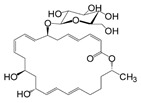	**FabG**	*S. aureus*	100	[17]
*NAS-21*	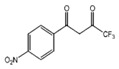	**FabZ**	*M. smegmatis*	360	[18]
*NAS-91*	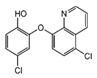	**FabZ**	*M. smegmatis*	498	[18]
*emodin*	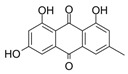	**FabZ**	*F. tularensis*	43.1 ± 9.2	[19]
			*Y. pestis*	29.7 ± 6.0	[19]
			*H. pylori*	9.70 ± 1.0	[20]
*mangostin*	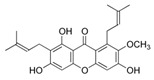	**FabZ**	*F. tularensis*	7.7 ± 2.0	[19]
			*Y. pestis*	6.1 ± 1.4	[19]
*stictic acid*	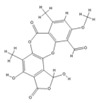	**FabZ**	*F. tularensis*	27.8 ± 6.1	[19]
			*Y. pestis*	13.0 ± 1.4	[19]
*1,4-naphthoquinone*	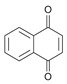	**FabD**	*M. catarrhalis*	23.18 ± 2.48	[21]
		**FabZ**	*M. catarrhalis*	26.67 ± 3.34	[21]
*juglone*	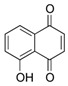	**FabD**	*H. pylori*	20 ± 1	[22]
		**FabZ**	*F. tularensis*	5.4 ± 1.4	[19]
			*Y. pestis*	5.3 ± 1.0	[19]
			*H. pylori*	30 ± 4	[22]
*triclosan*	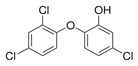	**FabI**	*E. coli*	0.98	[23]
			*S. aureus*	0.44–0.66	[23,24,25]
			*P. falciparum*	0.05–2	[15,26]
			*C. trachomatis*	0.32 ± 0.08	[27]
*AFN-1252*	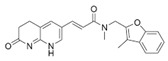	**FabI**	*C. trachomatis*	0.95 ± 0.21	[27]
*xanthorrhizol*	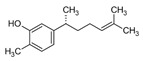	**FabI**	*E. coli*	17.1 ± 1.8	[28]
*complestatin*	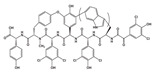	**FabI**	*S. aureus*	0.5	[25]
		**FabK**	*S. pneumoniae*	10	[25]
*neuroprotectin A*	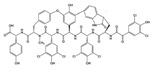	**FabI**	*S. aureus*	0.3	[25]
*chloropeptin I*	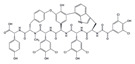	**FabI**	*S. aureus*	0.6	[25]
*meleagrin*	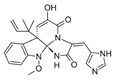	**FabI**	*S. aureus*	40.1	[23]
			*E. coli*	33.2	[23]
*phellinstatin*	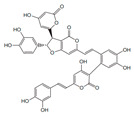	**FabI**	*S. aureus*	6	[29]
*chalcomoracin*	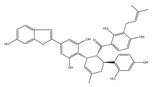	**FabI**	*S. aureus*	5.5	[30]
*moracin C*	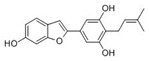	**FabI**	*S. aureus*	83.8	[30]
*aquastatin A*	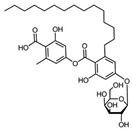	**FabI**	*S. aureus*	3.2	[31]
		**FabK**	*S. pneumoniae*	9.2	[31]
*atromentin*	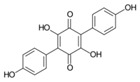	**FabK**	*S. pneumoniae*	0.24	[32]
*leucomelone*	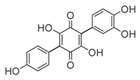	**FabK**	*S. pneumoniae*	1.57	[32]
*(Z)-1-oxooctadec-11-enylphosphoramidic acid*	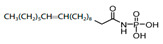	**PlsY**	*S. pneumoniae*	11	[33]
*1,1-difluoro-2-oxotridecylphosphonic acid*	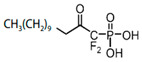	**PlsY**	*B. anthracis*	25	[33]
*phenyl (8-phenyloctanoyl) sulfamate*	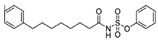	**PlsY**	*S. aureus*	25	[34]
*dioctylamine*	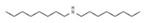	**CfaS**	*H. pylori*	63.81	[35]

IC_50_ is determined as concentration required for 50% inhibition of activity of appropriate enzyme.

**Table 2:** Major inhibitors of lipid A biosynthetic pathway.

Inhibitor	Structure	Enzyme	Origin	IC_50_, μM	References
*peptide 920*	NH_2_-SSGWMLDPIAGKWSR-COOH	**LpxA**	*E. coli*	0.06 ± 0.01	[206]
*RJPXD33*	TNLYMLPKWDIP-NH_2_	**LpxA**	*E. coli*	19.0 ± 1.2	[207]
		**LpxD**	*E. coli*	3.5 ± 0.1	[207]
*(R)-(3-(2-chloro-6-methoxybenzyl)morpholino)(3-(4-methylpyridin-2-yl)-1H-pyrazol-5-yl)methanone*	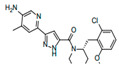	**LpxA**	*E. coli*	0.6	[208]
*BB-78485*	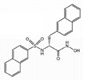	**LpxC**	*E. coli*	0.16 ± 0.07	[209]
*L-161,240*	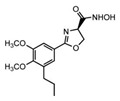	**LpxC**	*E. coli*	0.023 ± 0.003	[210]
			*P. aeruginosa*	0.22 ± 0.003	[210]
*L-573,655*	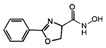	**LpxC**	*E. coli*	8.5	[211]
*CHIR-090*	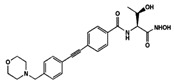	**LpxC**	*A. aeolicus*	~0.003	[212]
			*E. coli*	0.009	[213]
			*R. leguminosarum*	0.69	[213]
*LpxC-4*	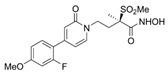	**LpxC**	*P. aeruginosa*	0.001	[214]
			*K. pneumoniae*	0.00007	[214]
			*A. baumannii*	0.183	[214]
*TU-514*	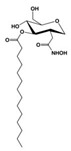	**LpxC**	*A. aeolicus*	7.0 ± 0.5	[215]
			*E. coli*	7.2 ± 1.9	[215]
*4-(2-Chlorophenyl)-3-hydroxy-7,7-dimethyl-2-phenyl-6,7,8,9-tetrahydro-2H-pyrazolo[3,4-b]quinolin-5(4H)-one*	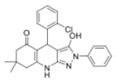	**LpxD**	*E. coli*	3.2	[216]
*1-(5-((4-(3-(trifluoromethyl)phenyl)piperazin-1-yl)sulfonyl)indolin-1-yl)ethan-1-one*	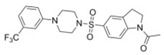	**LpxH**	*E. coli*	1.2 ± 0.2	[217]
*AZ1*	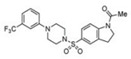	**LpxH**	*K. pneumoniae*	0.36	[218]
			*E. coli*	0.14	[218,219]
*JH-LPH-28*	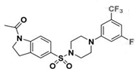	**LpxH**	*K. pneumoniae*	0.11	[218]
			*E. coli*	0.083	[218]
*JH-LPH-33*	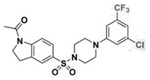	**LpxH**	*K. pneumoniae*	0.026	[218]
			*E. coli*	0.046	[218]

**Table 3:** The effects of antibacterial agents on the model lipid membrane’s permeability.

Agent	Structure	*C_min_*, μM	*C_tr_*, μM	Lipid Composition	References
Pore formation					
*gramicidin A*	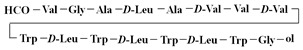	0.001	– *	DSPC	[254]
*alamethicin*	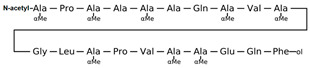	0.1	– *	DOPS:DOPE 1:1 (m/m)	[293]
*pardaxin*	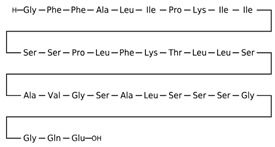	0.006	– *	soybean lecithin	[258]
*melittin*	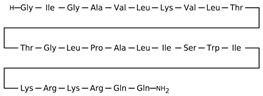	0.23	– *	POPC:cholesterol 3:1 (m/m)	[294]
*magainin I*	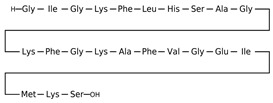	10	– *	DOPC; DOPE:DOPG 3:1 (m/m)	[260]
*magainin II*	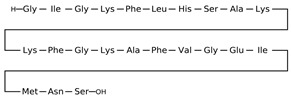	0.08	– *	POPC:DOPG 6:1 (m/m); DOPS:ergosterol 3:1 (m/m); POPC:ergosterol 3:1 (m/m)	[261]
*mastoparan*	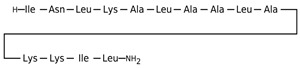	0.68	– *	DPhPC	[262]
*ceratotoxin A*	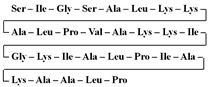	0.02	– *	POPC:DOPE 7:3 (*w*/*w*); POPC:DOPE:POPS 7:3:1 (*w*/*w*)	[271,272]
*protegrin-1*	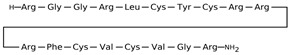	0.25–10	– *	DOPC:DOPE 1:1 (m/m)	[259]
*nisin*	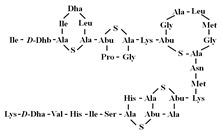	0.1		lipid II	[295]
		~40	>500	TOCL	[265]
*cinnamycin*	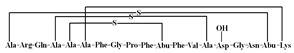	~1.5	>10	DOPE; TOCL	[265]
*duramycin*	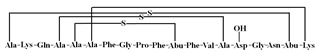	4.5–10	– *	GMO	[266]
		~2	>12	DOPE; TOCL	[265]
*rabbit α-defensins (NP-1/2)*	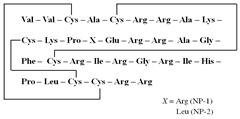	~1	>16	PE/PC/PS 2:2:1 (*w*/*w*); PE/CL	[296,297]
*daptomycin*	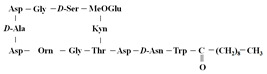	6.2	– *	DPhPG	[298]
*polymyxin B*	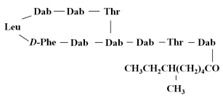	2.5	>100	DOPG	[108]
		1	>20	Kdo_2_-Lipid A	
*gaysemycin*	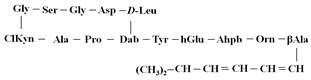	~26	– *	DOPG	[279]
Pore formation and detergent action					
*cecropin A*	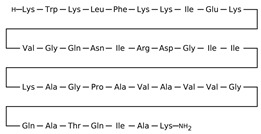	1	>5	DOPS:DOPE 1:1 (m/m)	[264]
*cecropin B*	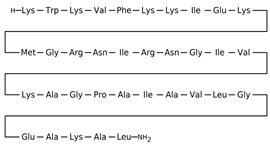	1	>5	DOPS:DOPE 1:1 (m/m)	[264]
Detergent action					
*cecropin P1*	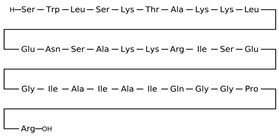	– *	>50	DOPS:DOPE 1:1 (m/m)	[264]
*aurein 1.2*	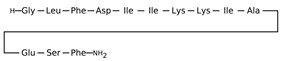	– *	>10	DMPG	[287]

*C_min_*—the antibiotic threshold concentration required to observe single pores; *C_tr_*—the antibiotic threshold concentration required to disintegrate the lipid bilayers; *—data are absent. Abbreviations: DSPC—1,2-distearoyl-*sn*-glycero-3-phosphocholine; DPPC—1,2-dipalmitoyl-*sn*-glycero-3-phosphocholine; POPC—1-palmitoyl-2-oleoyl-*sn*-glycero-3-phosphocholine; DOPC—1,2-dioleoyl-*sn*-glycero-3-phosphocholine; DOPE—1,2-dioleoyl-*sn*-glycero-3-phosphoethanolamine; DOPG—1,2-dioleoyl-*sn*-glycero-3-phospho-(1′-rac-glycerol); DOPS—1,2-dioleyl-*sn*-glycero-3-phosphoserine; DPhPC—1,2-diphytanoyl-*sn*-glycero-3-phosphocholine; DPhPG—1,2-diphytanoyl-*sn*-glycero-3-phospho-(1′-rac-glycerol); DMPG—1,2-dimyristoyl-*sn*-glycero-3-phospho-(1′-rac-glycerol); TOCL—1′,3′-bis-[1,2-dioleoyl-*sn*-glycero-3-phospho]-1′,3′-glycerol; GMO—glyceryl monooleate; Kdo_2_-Lipid A—di [3-deoxy-D-manno-octulosonyl]-lipid A. Abbreviations for nonproteinogenic amino acids: α-Me-Ala—α-methyl-alanyl; Phe-ol—phenylalaninol; Dab—2,4-diaminobutyric acid; Dhb—2,3-dehydroaminobutyric acid; Dhb—2,3-didehydrobutyrine, Abu—α-aminobutyric acid; MeOGlu—3-methyl-glutamic acid; Kyn—kynurenine; Orn—ornithine. Only D-enantiomers of amino acids are indicated.

**Table 4:** Major inhibitors of fungal sphingolipid biosynthesis.

Inhibitor	Structure	Enzyme		IC_50_, μM	References
*sphingofungin B*	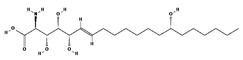	**SPT**	*C. albicans*	0.049	[350]
			*S. cerevisiae*	0.051	
*viridiofungin A*	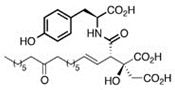	**SPT**	*C. albicans*	0.022	[350]
			*S. cerevisiae*	4.7	
*viridiofungin B*	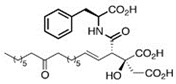	**SPT**	*C. albicans*	0.017	[350]
			*S. cerevisiae*	1.84	
*viridiofungin C*	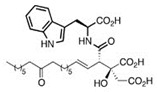	**SPT**	*C. albicans*	0.025	[350]
			*S. cerevisiae*	1.68	
*aureobasidin A*	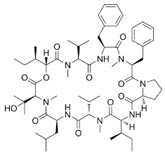	**IPCS**	*C. albicans*	0.002	[363]
			*C. glabrata*	0.002	
			*Candida tropicalis*	0.003	
			*Candida parapsilosis*	0.003	
			*Candida krusei*	0.003	
			*A. fumigatus*	0.005	
			*Aspergillus flavus*	0.002	
			*Aspergillus terreus*	0.004	
			*Aspergillus niger*	0.004	
			*S. cerevisiae*	0.0009	[358]
*khafrefungin*	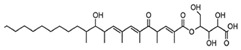	**IPCS**	*C. albicans*	0.0006	[356]
			*C. neoformans*	0.031	
			*S. cerevisiae*	0.007	
*haplofungin A*	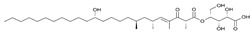	**IPCS**	*S. cerevisiae*	0.0025	[357]
			*A. fumigatus*	0.41	
*haplofungin B*	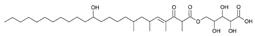	**IPCS**	*S. cerevisiae*	0.042	[357]
			*A. fumigatus*	1.33	
*pleofungin A*	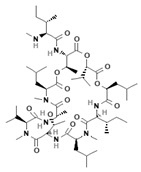	**IPCS**	*S. cerevisiae*	0.007	[358]
			*A. fumigatus*	0.0009	
*galbonolide A (rustmicin)*	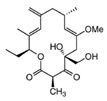	**IPCS**	*C. albicans*	0.0038	[359]
			*C. neoformans*	0.00007	
			*S. cerevisiae*	0.0198	

**Table 6:** The effects of antifungal agents on the model lipid membrane’s permeability.

Agent	Structure	C_min_, μM	C_tr_, μM	Target Lipid	References
Pore formation					
*syringomycin E*	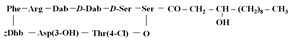	1–5	– *	DPhPC; DOPS:DOPE 1:1 (m/m)	[422]
*syringopeptin 22A*	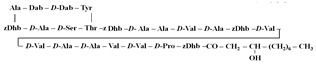	0.003	– *	DPhPC; DOPS:DOPE 1:1 (m/m)	[416]
*syringopeptin 25A*	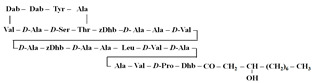	0.004	– *	PC:PE:PS 2:2:1 (m/m/m)	[428]
*fengycins*	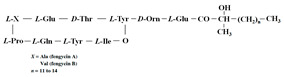	0.1–0.5	>10	POPC:POPE:POPG:ergosterol 2:2:5:1 (m/m)	[425]
*surfactin*	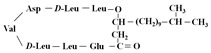	0.2–0.4	– *	DPhPC	[424]
		1.4	– *	glyceryl monooleate	[426]
*iturin A*	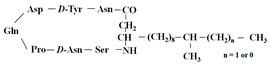	0.001	– *	egg-PC; egg-PC:DMPE 8:2 (*v*/*v*)	[429]
		– *	– *	glyceromonoolein	[421]
*mycosubtilin*	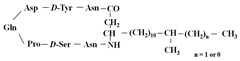	– *	–*	DPhPC	[430]
*bacillomycins*	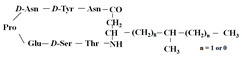	– *	– *	glyceromonoolein	[421]
*amphotericin B*	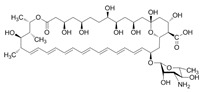	0.02–0.03	– *	phospholipid:cholesterol 20:1 (m/m)	[431]
		0.01	>20	DPhPC:ergosterol 2:1 (m/m)	[432]
*nystatin*	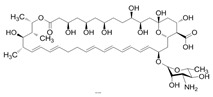	0.1	– *	phospholipid:cholesterol 20:1 (m/m)	[431]
		0.01	>100	DPhPC:ergosterol 2:1 (m/m)	[433]
*filipin*	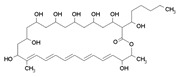	0.02	– *	phospholipid:cholesterol 2:1 (*v*/*v*)	[434]
		0.01	>100	DPhPC:ergosterol 2:1 (m/m)	[435]
*piscidin*	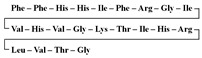	0.005	– *	azolectin	[436]
Detergent action					
*natamycin*	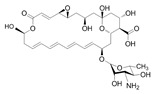	– *	110	DPhPC:ergosterol 2:1 (m/m)	[437]

*C_min_*—the antibiotic threshold concentration required to observe single pores; *C_tr_*—the antibiotic threshold concentration required to disintegrate the lipid bilayers; *—data are absent. Abbreviations: DPhPC—1,2-diphytanoyl-*sn*-glycero-3-phosphocholine; DOPS—1,2-dioleoyl-*sn*-glycero-3- phosphoserine; DOPE—1,2-dioleoyl-*sn*-glycero-3-phosphoethanolamine; POPC—1-palmitoyl-2-oleoyl-*sn*-glycero-3-phosphocholine; POPE—1-palmitoyl-2-oleoyl-*sn*-glycero-3-phosphoethanolamine; POPG—1-palmitoyl-2-oleoyl-*sn*-glycero-3-phospho-(1′-rac-glycerol); DOPG—1,2-dioleoyl-*sn*-glycero-3-phospho-(1′-rac-glycerol); DMPE—1,2-dimyristoyl-*sn*-glycero-3-phosphoethanolamine. Abbreviations for nonproteinogenic amino acids: α-Me-Ala—α-methyl-alanyl; Phe-ol—phenylalaninol; Dab—2,4-diaminobutyric acid; Dhb—2,3-dehydroaminobutyric acid; Dhb—2,3-didehydrobutyrine; Abu—α-aminobutyric acid; MeOGlu—3-methyl-glutamic acid; Kyn—kynurenine; Orn—ornithine. Only D-enantiomers of amino acids are indicated.

**Table 8:** Molecular tweezers and their antiviral activity.

Molecular Tweezer	Structure	Virus	IC_50_, µM	Reference
*CLR01*	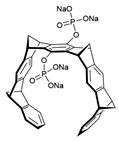	HIV-1	13.7–20.1	[501]
		Ebola	25.8	[502]
		Zika	8.2	[502]
		HSV-1	19.3	[500]
		HSV-2		
		measles		
		IVA		
		SARS-CoV-2	76.7	[503]
*CLR05*	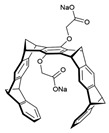	Zika	38.1	[500]
		HSV-1		
		HSV-2		
		measles		
		influenza		
		SARS-CoV-2	167.3	[503]
*CLR01e*	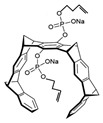	HIV-1	~10	[500]
*CLR01f*	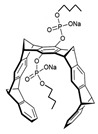	HIV-1	~7	[500]
*CP006*	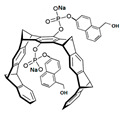	SARS-CoV-2	0.3	[503]
*CP020*	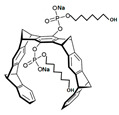	SARS-CoV-2	0.4	[503]
*CP025*	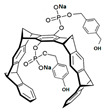	SARS-CoV-2	0.6	[503]
*CP036*	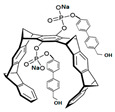	SARS-CoV-2	0.2	[503]

**Table 9:** Antimicrobial peptides with direct antiviral action through lipid envelope disruption.

Antimicrobial Peptide	Structure	Virus	IC_50_, µM	Reference
*D-plantaricin NC8 α*	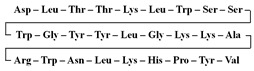	SARS-CoV-2	~0.001	[507]
		IVA	~0.1	[507]
*kalata B1*	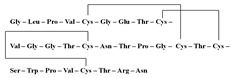	HIV	~2–5	[508]

**Table 10:** Fusion inhibitors affecting membrane curvature stress and their antiviral activity.

Inhibitor	Structure	Virus	IC_50_, µM	Reference
*aculeacin A*	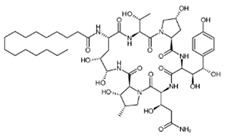	SARS-CoV-2	1.3 ± 0.3	[517]
*anidulafungin*	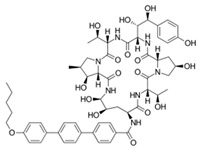	SARS-CoV-2	4.7 ± 0.9	[517]
*iturin A*	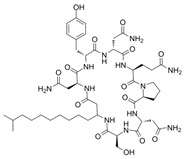	SARS-CoV-2	10.9 ± 2.0	[517]
*mycosubtilin*	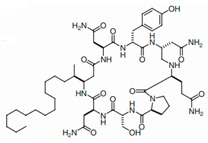	SARS-CoV-2	2.3 ± 0.3	[517]
